# Disproportionate Contributions of Select Genomic Compartments and Cell Types to Genetic Risk for Coronary Artery Disease

**DOI:** 10.1371/journal.pgen.1005622

**Published:** 2015-10-28

**Authors:** Hong-Hee Won, Pradeep Natarajan, Amanda Dobbyn, Daniel M. Jordan, Panos Roussos, Kasper Lage, Soumya Raychaudhuri, Eli Stahl, Ron Do

**Affiliations:** 1 Center for Human Genetic Research, Massachusetts General Hospital, Boston, Massachusetts, United States of America; 2 Cardiovascular Research Center, Massachusetts General Hospital, Boston, Massachusetts, United States of America; 3 Department of Medicine, Harvard Medical School, Boston, Massachusetts, United States of America; 4 Program in Medical and Population Genetics, Broad Institute, Cambridge, Massachusetts, United States of America; 5 Icahn Institute for Genomics and Multiscale Biology, Department of Genetics and Genomics, Icahn School of Medicine at Mount Sinai, New York, New York, United States of America; 6 Charles Bronfman Institute for Personalized Medicine, Department of Genetics and Genomic Sciences, Icahn School of Medicine at Mount Sinai, New York, New York, United States of America; 7 Division of Psychiatric Genomics, Icahn School of Medicine at Mount Sinai, New York, New York, United States of America; 8 Department of Surgery, Massachusetts General Hospital, Boston, Massachusetts, United States of America; 9 Analytic and Translational Genetics Unit, Department of Medicine, Massachusetts General Hospital, Boston, Massachusetts, United States of America; 10 Division of Rheumatology, Immunology, and Allergy, Brigham and Women's Hospital and Harvard Medical School, Boston, Massachusetts, United States of America; 11 Faculty of Medical and Human Sciences, University of Manchester, Manchester, United Kingdom; 12 Center for Statistical Genetics, Department of Genetics and Genomic Sciences, Icahn School of Medicine at Mount Sinai, New York, New York, United States of America; 13 Zena and Michael A. Weiner Cardiovascular Institute, Icahn School of Medicine at Mount Sinai, New York, New York, United States of America; Stanford University School of Medicine, UNITED STATES

## Abstract

Large genome-wide association studies (GWAS) have identified many genetic loci associated with risk for myocardial infarction (MI) and coronary artery disease (CAD). Concurrently, efforts such as the National Institutes of Health (NIH) Roadmap Epigenomics Project and the Encyclopedia of DNA Elements (ENCODE) Consortium have provided unprecedented data on functional elements of the human genome. In the present study, we systematically investigate the biological link between genetic variants associated with this complex disease and their impacts on gene function. First, we examined the heritability of MI/CAD according to genomic compartments. We observed that single nucleotide polymorphisms (SNPs) residing within nearby regulatory regions show significant polygenicity and contribute between 59–71% of the heritability for MI/CAD. Second, we showed that the polygenicity and heritability explained by these SNPs are enriched in histone modification marks in specific cell types. Third, we found that a statistically higher number of 45 MI/CAD-associated SNPs that have been identified from large-scale GWAS studies reside within certain functional elements of the genome, particularly in active enhancer and promoter regions. Finally, we observed significant heterogeneity of this signal across cell types, with strong signals observed within adipose nuclei, as well as brain and spleen cell types. These results suggest that the genetic etiology of MI/CAD is largely explained by tissue-specific regulatory perturbation within the human genome.

## Author Summary

Coronary artery disease (CAD) and its subcomponent, myocardial infarction (MI), are the leading causes of infirmity and death worldwide. Large-scale genetic association studies have identified many genetic markers associated with CAD and MI. However, it has been difficult to determine the precise functional effects of these markers. Furthermore, it is unknown which cell types are biologically important in the development of MI/CAD. By intersecting findings from large-scale genetic association studies with functional genomic annotations, we show that genetic markers located in genomic regions that regulate expression of genes make up a large proportion of the genetic risk of MI/CAD. Furthermore, we show that this effect is particularly strong in certain tissues, including adipose, brain and spleen tissue. These results highlight the role of tissue-specific regulatory mechanisms in the genetic etiology of MI/CAD.

## Introduction

Coronary artery disease (CAD) and myocardial infarction (MI) remain among the leading causes of infirmity and death worldwide despite advances in and widespread adoption of medical therapies treating this disease. Studies have shown a large genetic component for CAD, with the heritability estimated to be 30–60% [1]. Large-scale genome-wide association studies (GWAS) have identified common single nucleotide polymorphisms (SNPs) at 45 loci associated with MI/CAD risk [2–8]. Although these newly discovered loci have led to important new biological insights for MI/CAD [9,10], the proportion of the heritability explained by these loci represents approximately 15% of the estimated heritability [8]. Therefore, a large proportion of the genetic effects are apparently not explained by known loci. This phenomenon has been similarly observed with GWAS for other complex diseases [11].

Previously, we modeled the genetic architecture of MI and CAD using GWAS data [12]. Using simulated genetic models, we inferred that a polygenic model comprised of thousands of associated common variants with small effects explains the majority of the heritability (proportion of total liability-scale variance explained is 0.48, 76% of family-study h^2^) for MI/CAD [12]. Furthermore, recent work has partitioned heritability of complex diseases into broad categories of the genome [13]. Pooling 11 common diseases, including CAD, Gusev et al. have shown that heritability is disproportionally represented in regulatory elements, specifically in DNase I hypersensitivity sites (DHS) (h^2^_g_ = 79% for SNPs in DHS regions). However, a specific analysis of CAD yielded a wide interval of the true enrichment of DHS, with genotyped estimates of h^2^_g_ = 10.9 to 71.3% (95% confidence intervals) for SNPs in DHS regions. Hence, other than being broadly distributed throughout the genome, the molecular consequences of MI/CAD-associated common variants largely remain undefined. Furthermore, it is unclear which cell or tissue types are influenced the most by MI/CAD-associated SNPs.

We addressed these unresolved issues by leveraging data from the National Institutes of Health (NIH) Roadmap Epigenomics Project [14,15] and the Encyclopedia of DNA Elements (ENCODE) Consortium [16]. These projects have comprehensively catalogued biochemical functional regions such as those critical for transcription, transcription factor binding, chromatin structure and histone modification in different cell types, providing unique opportunities to scrutinize links between non-protein-coding DNA sequence variants and gene function. Studies have shown that SNPs in these functional DNA elements can regulate gene expression [17] and common disease-associated loci [18,19].

Using these data, we partitioned the genetic risk of MI/CAD into different categories, to discern drivers in specific cell types that may biologically influence MI/CAD. First, we investigated components of polygenicity and heritability in distinct genomic compartments. Second, we tested for differences in polygenicity, enrichment measures and heritability across diverse cell types within three histone modification marks. Third, we examined clusters of 45 loci discovered from recent GWAS meta-analyses [8] for MI/CAD mapping to the three histone marks in different cell types. Finally, we investigated whether specific biological networks were expressed differently in certain cell types.

## Results

### Relative contribution of genomic compartments to MI/CAD risk

We imputed two GWAS datasets, the Myocardial Infarction Genetics Consortium (MIGen) and the Wellcome Trust Case Control Consortium (WTCCC) CAD, using reference haplotypes from the 1000 Genomes Project [20]. We imputed ~7 million SNPs with a high imputation quality metric (>0.5) in both datasets. First, we investigated the relative contributions of different genomic compartments to MI/CAD risk. We partitioned the human genome into three distinct variant sets: “genic noncoding”, “genic coding” and “intergenic” (S1 Table) (see Materials and Methods). For each variant set, we performed two different analyses: 1) polygenic risk score analysis, where we test association of a genetic score comprised of multiple SNPs and 2) SNP-heritability analysis, where we estimate the variance in liability to MI/CAD [21] (see Materials and Methods).

**Fig 1:**
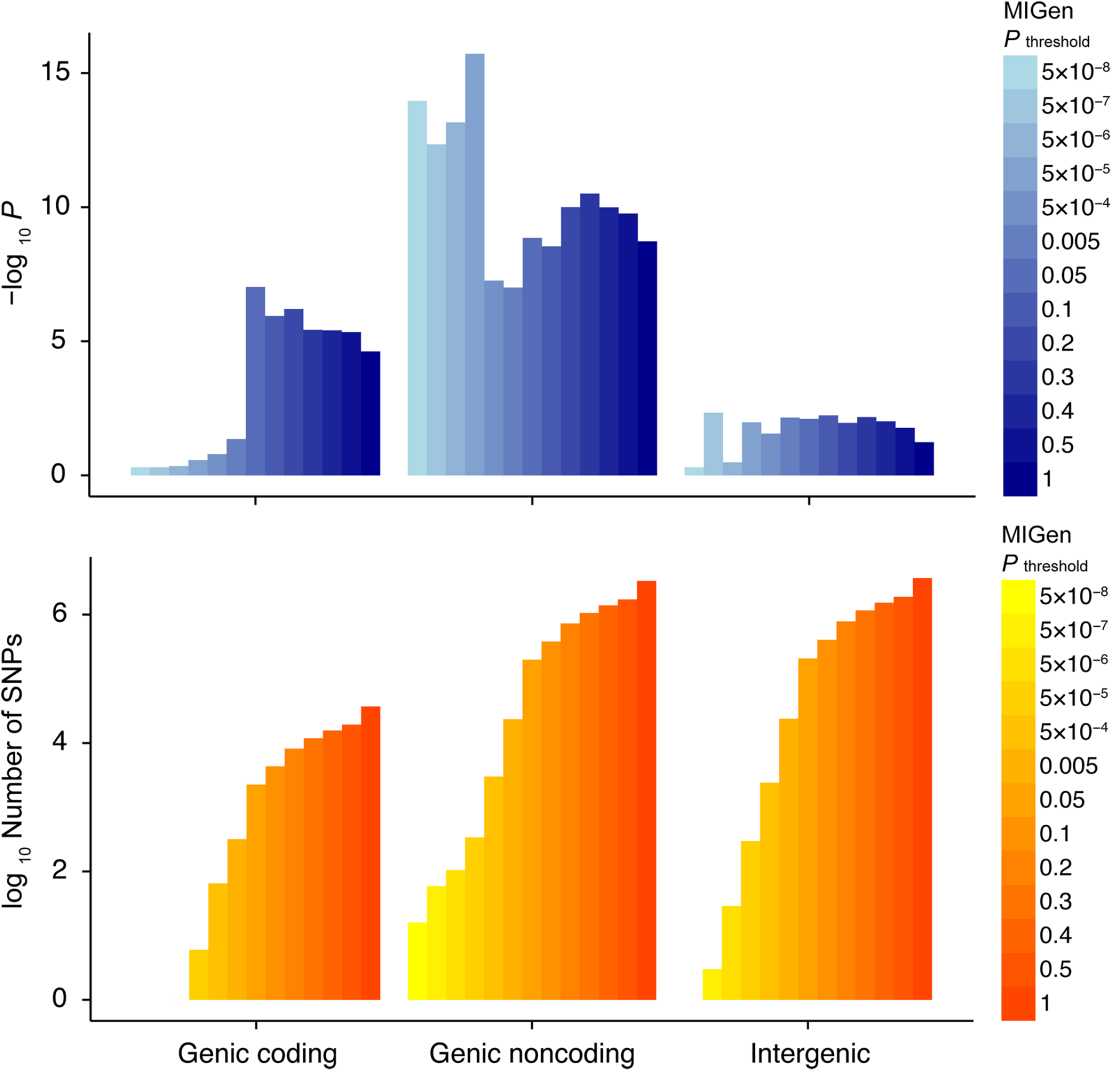
Contributions of three genomic compartments to the polygenicity of MI/CAD. Polygenic risk score analysis was performed across three different genomic compartments. The top bar plot represents the strength of association for the polygenic risk score analysis whereas the bottom bar plot represents the number of SNPs within each of the compartments. The strongest polygenic association signals were within noncoding regions adjacent to protein-coding genes (“genic noncoding”). MI, myocardial infarction; CAD, coronary artery disease; SNP, single nucleotide polymorphism. Genic coding, variants that code amino acid sequence within ±10 kilobases of the 3′ or 5′ untranslated regions of a gene. Genic noncoding, variants that do not code amino acid sequence within ±10 kilobases of the 3′ or 5′ untranslated regions of a gene. Intergenic, variants that are beyond ±10 kilobases of the 3′ or 5′ untranslated regions of a gene.

In the polygenic risk score analysis, we observed that the ‘genic’ variant set, defined as genomic regions within 10 kilobases (kb) upstream and downstream of a gene, showed a substantially stronger signal than SNPs in intergenic regions (defined as genomic regions outside of 10 kb of a gene) (Fig 1 and S1 Fig). Similar results were observed amongst genomic regions with window sizes of 20 kb and 50 kb (S2 and S3 Figs). Among this set of variants, both association (Fig 1) and the phenotypic variance explained by the polygenic risk scores before and after normalization by the number of SNPs (S1–S3 Figs) were the most significant in regions adjacent to the protein-coding DNA regions, called “genic noncoding” regions. We observed that polygenic risk scores were strongly associated with MI/CAD in “genic noncoding” regions (*P*<10^−10^), explaining 1 to 1.5% of the phenotypic variance. By comparison, polygenic risk scores in regions within protein-coding DNA regions, called “genic coding” regions, were less strongly associated (*P*<10^−5^) and explained approximately 0.5% of the variability. Similar patterns were observed after normalizing by the number of SNPs in the polygenic risk score. These patterns were particularly evident for *P* value thresholds <10^−5^ (see Materials and Methods). The association signal remained among the variant sets with discovery *P* value thresholds greater than or equal to 0.05 after excluding regions within ±1 megabase of the 45 known SNPs associated with MI/CAD risk [8] (S4 Fig).

We further examined the role of different genomic compartments on the heritability for MI/CAD risk by testing whether SNPs in the three compartments make up a large portion of the heritability for MI/CAD (Table 1 and S2 Table). Consistent with findings from our polygenic risk score analysis, we observed that most of the heritability resides within the “genic” regions. In a meta-analysis of the MIGen and WTCCC CAD studies, SNPs in “genic noncoding” regions explained approximately 58.9% (variance in liability = 0.25, *P* = 1×10^−9^) of the total heritability, resulting in a fold enrichment of variance of 1.2. In contrast, the heritability of MI/CAD explained by SNPs residing within “genic coding” regions was estimated to be only 10% of the total (variance in liability = 0.042, *P* = 0.07). SNPs residing within “genic coding” regions accounted for only 0.5% of the total number of variants, resulting in a high fold enrichment of variance of 19.1. Despite the enrichment of variance estimates for both “genic coding” and “genic noncoding”, neither category statistically deviated beyond expectation (*P* = 0.088 and *P* = 0.23 respectively). On the other hand, a statistically significant depletion of variance in liability was observed in the “intergenic” regions compared to expectation (observed variance in liability = 0.13, expected variance in liability = 0.22 [0.52 fraction of SNPs of total × 0.42 total variance in liability], difference in observed and expected variance in liability *P* = 0.0089). Similar results were observed amongst genomic regions with window sizes of 20 kb and 50 kb (S3 and S4 Tables). Heritability estimates with a prevalence of 3% of early-onset MI/CAD showed reduced heritability (variance in liability = 0.35) explained by the genomic compartment whereas *P* values and enrichment of variance remained the same (S5 Table).

**Table 1:** Heritability of MI/CAD explained by three genomic compartment sets. We calculated the SNP-heritability in three genomic compartment sets for MI/CAD in a meta-analysis of the MIGen and WTCCC CAD studies using the Genome-wide Complex Trait Analysis (GCTA) software. We observed increased enrichment in variance in both “genic coding” and “genic noncoding” regions.

Genomic compartments	Variance^1^	V-SE^1^	*V-P* ^1^	Number of SNPs	% Variance of total	% SNPs of total	Enrichment of variance^2^	Deviation from expected variance *P* ^3^
Genic coding	0.042	0.023	0.07	37,142	10.0	0.5	19.1	0.088
Genic noncoding	0.25	0.041	1×10^−9^	3,355,483	58.9	47.3	1.2	0.23
Intergenic	0.13	0.034	0.0001	3,703,319	31.1	52.2	0.6	0.0089
Whole genome as sum	0.42			7,095,944	100.0	100.0	1.0	

Heritability estimates were inferred independently first in MIGen and WTCCC CAD from a single model involving three variance components (“genic coding”, “genic noncoding” and “intergenic”) using the GCTA software [22,23]. Heritability estimates shown here are from a meta-analysis of the Variance and standard error (V-SE) from these models using as weights the inverse variance from these models.

^1^Variance and V-SE are estimates from the ratio of genetic variance to phenotypic variance for the specified variance component whereas the *P* value (V-P) is from the likelihood ratio test of a reduce model with the specified genetic variance component dropped from the full model, from the restricted maximum likelihood method in the GCTA software [22,23].

^2^Enrichment of variance was calculated as the % variance of total divided by % SNPs of total. MI, myocardial infarction; CAD, coronary artery disease; SNP, single nucleotide polymorphism.

^3^ *P* value from difference in the observed variance minus the expected variance (variance of whole genome as sum multiplied by % SNPs of total). Genic coding, variants that code amino acid sequence within ±10 kilobases of the 3′ or 5′ untranslated regions of a gene. Genic noncoding, variants that do not code amino acid sequence within ±10 kilobases of the 3′ or 5′ untranslated regions of a gene. Intergenic, variants that are beyond ±10 kilobases of the 3′ or 5′ untranslated regions of a gene.

### Strength of polygenic association signal within regulatory elements

Given the high polygenicity for MI/CAD explained by SNPs in noncoding regions surrounding protein-coding regions, we further examined whether polygenicity is stronger within specific regulatory elements. Using data from the NIH Roadmap Epigenomics Project (see **URL**), we specifically examined three histone modification marks that are indicative of active promoters (H3K4me3/H3K9ac) or enhancers (H3K27ac). A polygenic association signal comprised of SNPs with association *P*<0.05 for MI/CAD was stronger in the histone marks, beyond what we expect by chance after randomly sampling “genic noncoding” regions outside of the marks (Mann-Whitney test *P* = 1.1×10^−95^) (S5 Fig).

### Genetic contribution of regulatory elements between specific cell types

**Fig 2:**
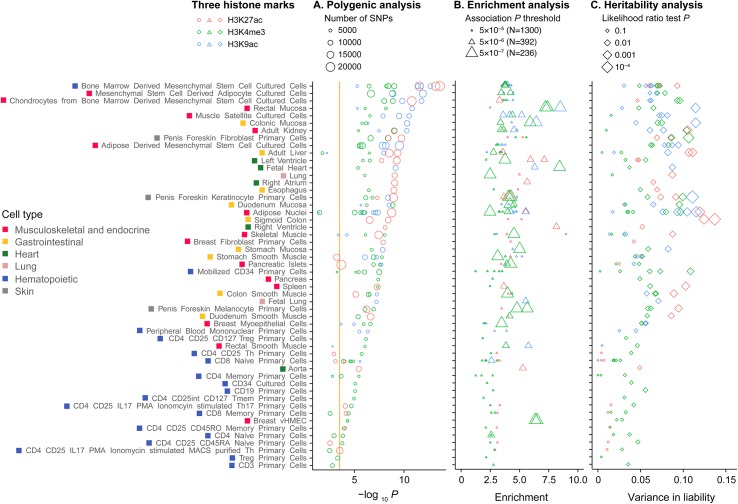
Polygenic, enrichment and heritability analysis of three histone modification marks across cell types. We performed three different analyses to test for cell type specific effects on the genetic risk for MI/CAD. Analyses were conducted on SNPs residing in the three histone marks (H3K27ac, H3K4me3, H3K9ac) that were present in the different cell types. (A) Polygenic risk score analysis. We performed polygenic risk score association analysis on SNPs with MIGen discovery association *P*<0.05. Negative logarithm of *P* values from association testing of the polygenic risk score performed in the WTCCC CAD was shown. Cell types were sorted based on the strength of polygenic association. Orange vertical line represents a significant level with 5% alpha error. (B) Enrichment of association. Enrichment analyses were performed by comparing the proportion of significant variants passing a specific association *P* threshold of a variant set with that of a baseline set. Different association *P* thresholds 5×10^−7^, 5×10^−6^, 5×10^−5^ from the CARDIoGRAM study were tested [24]. The variant sets in this analysis were SNPs in the specified histone marks that were present in the indicated cell type. For the baseline set, we test SNPs in regions that are outside of these histone marks within 10 kilobases (kb) of the protein coding regions of the genome. To reduce the effects of linkage disequilibrium, these baseline SNPs were selected to be 5 kb away from the histone marks. In the plot, each triangular point represents the strongest enrichment result for each mark in each cell type across the three possible association *P* thresholds. (C) Heritability analysis. Heritability analysis was performed within histone marks in the MIGen study. Each point in the plot represents the variance in liability generated from a joint model involving two variance components using the Genome-wide Complex Trait Analysis software [22,23]. The two variance components include 1) SNPs in the specified histone mark that was present in the indicated cell type and 2) all other SNPs outside of these regions. The variance in liability is an estimate from the ratio of genetic variance to phenotypic variance for the specified variance component (i.e. the specified variance component is all SNPs within the specified histone mark) whereas the *P* value is from the likelihood ratio test of a reduce model with the specified genetic variance component dropped from the full model, from the restricted maximum likelihood method in the Genome-wide Complex Trait Analysis software [22,23]. MI, myocardial infarction; CAD, coronary artery disease; SNP, single nucleotide polymorphism.

We next tested for differences in polygenicity, enrichment and heritability estimates between different cell or tissue types within the three histone modification marks (Fig 2). We observed heterogeneity on the polygenicity of MI/CAD between cell types (Fig 2A). For example, SNPs in the H3K27ac and H3K9ac in bone marrow derived mesenchymal stem cell cultured cells and SNPs in the H3K4me3 regions in mesenchymal stem cell derived adipocyte cultured cells were amongst the strongest signals. Meanwhile, SNPs in any of the three histone marks in hematopoietic CD3, Treg, CD4, CD25, CD45RA primary cells were amongst the weakest signals. Enrichment analyses showed strong excesses for highly associated SNPs (*P*<10^−5^), compared with matched random SNPs, for mesenchymal stem cells, heart tissues such as fetal heart and ventricle, and intestinal mucosa including rectal and colonic mucosa, among others (Fig 2B). Cell-type specific effects were also generally consistent for heritability estimates (Fig 2C). The cell-type specific enrichment signals were strongest among strongly associated SNPs (Fig 2B).

When tested for ENCODE data, statistically significant polygenicity and high heritability estimates were observed for variants residing in specific active chromatin regions, including enhancers and weak transcription regions (about 3 and 11% of reference genome, respectively) across several cell lines, including skeletal muscle and vascular cell lines (S6 Fig). Polygenicity and heritability estimates were substantially weaker for variants in inactive chromatin states than those in active states (S7 Fig), with the notable exception of the quiescent state. Heritability estimates for variants in a quiescent state were particularly high although the variants in this state accounted for >60% of the reference epigenome (S7 Fig). However, the enrichment of variance (% variance of total divided by % SNPs of total) in the quiescent state was low (on average 0.76 [0.50−0.92] in different cell lines) compared with those for enhancers (on average 6.5 [3.2−10.3]) and weak transcription (1.9 [1.1–2.8]). In general, we note that ENCODE cell line chromHMM state results were largely driven by the sizes of these genomic compartments (i.e. % SNPs), which would be expected if alignment of MI/CAD genetic effects and ChromHMM stats were random.

### Disproportionate number of MI/CAD-associated SNPs in regulatory elements in specific cell types

These observations have implications for fine-mapping loci discovered from large-scale GWAS. In particular, the results suggest that causal variants within GWAS loci are overrepresented in regulatory elements adjacent to protein-coding regions of the genome. Therefore, we investigated 45 independent, lead SNPs discovered from recent GWAS meta-analyses [8] for MI/CAD and tested whether a disproportionate number of these SNPs overlapped histone marks across diverse cell lines and tissues. We generated 10,000 random sets of SNPs that were selected to match the query GWAS SNPs based on similar minor allele frequency (±0.05 frequency), number of SNPs in linkage disequilibrium (LD) with the query SNP (±10% of number of SNPs in LD with query SNP using *r* ^2^>0.5), distance to nearest gene (±10% of distance of nearest gene from query SNPs) and gene density (±10% of number of genes in loci around the query SNPs) [25]. We excluded two SNPs (rs3798220 and rs12205331) out of the 45 GWAS SNPs because we were unable to find appropriate matching null SNPs. We observed that 25 (58.1%) out of 43 GWAS SNPs overlapped one of three histone marks. From the 10,000 random null sets, a median of 15 (34.9%) out of the 43 random SNPs (1^st^ quartile of 13 and 3^rd^ quartile of 17) overlapped histone marks (S8 Fig). Only a very small fraction of random sets showed a higher number of GWAS SNPs overlapping with histone marks than what we observed (4 out of 10,000 random null sets) (*P* = 4×10^−4^).

### Clustering of MI/CAD-associated SNPs in histone marks and cell types

**Fig 3:**
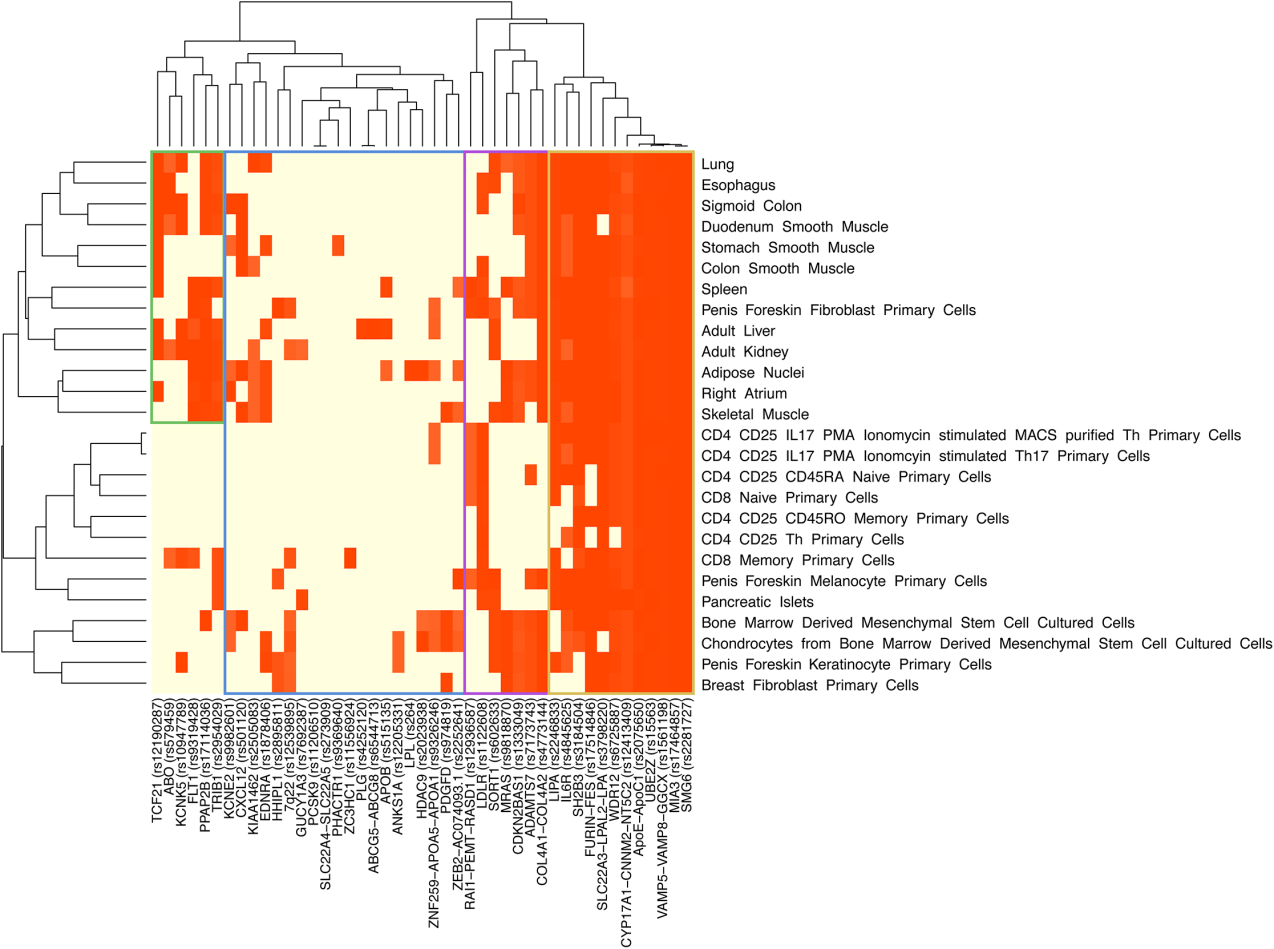
Hierarchical clustering of 45 MI/CAD GWAS SNPs and specific cell types for a histone modification mark (H3K27ac). We mapped 45 MI/CAD GWAS SNPs, as well as SNPs in high linkage disequilibrium (*r* ^2^≥0.8), to H3K27ac in different cell types. Hierarchical clustering was based on the presence or absence of a SNP residing in H3K27ac in different cell types and was performed using the heatmap function in R (R Project for Statistical Computing). We observed unique patterns between the different GWAS loci and cell types. For example, 12 of the 45 GWAS loci were expressed in more than 80% of the cell types, whereas 13 of the 45 GWAS loci were expressed in less than 20%. Red color indicates a lead SNP or tag SNPs (linkage disequilibrium value of *r* ^2^≥0.8) residing in H3K27ac in different cell types (See S9 and S10 Figs for H3K9ac and H3K4me3, respectively). MI, myocardial infarction; CAD, coronary artery disease; GWAS, genome-wide association study; SNP, single nucleotide polymorphism.

Because histone modification differed between cell types, we next examined whether the 45 MI/CAD GWAS SNPs yielded differential gene regulation across various tissues. We mapped the 45 GWAS SNPs, along with SNPs in high LD (*r* ^2^≥0.8), that reside within each of the three histone marks in different cell types. We observed distinct patterns between the different GWAS loci and cell types (Fig 3 and S9 and S10 Figs). For example, for the histone mark H3K27ac, 12 of the 45 loci were expressed in more than 80% of the cell types, whereas 13 of the 45 loci were expressed in less than 20%. Using HaploReg v2 [26], specific cell lines displayed enrichment with the 45 loci in strong enhancer regions. In particular, cell types related to adipose nuclei, spleen and brain tissue were amongst the strongest enrichment signals for inferred strong enhancer chromatin states (Table 2). We observed consistent results in these cell lines when also considering SNPs with less stringent significance levels using polygenic association analysis (Fig 2). HaploReg analysis for available ENCODE cell lines showed 24-fold enrichment with the 45 loci in enhancer regions in H1 cell lines (*P* = 3×10^−5^).

**Table 2:** Significant enrichment of 45 MI/CAD-associated SNPs in specific cell types detected by enrichment analysis. We examined whether 45 MI/CAD-associated loci were enriched in regions of inferred strong enhancer chromatin states [27] in specific cell types using NIH Roadmap data and two mammalian conservation algorithms, GERP and SiPhy-omega, implemented in HaploReg v2 [26]. We observed significant enrichment of MI/CAD-associated SNPs in specific cell types, including adipose nuclei, spleen and brain tissue. MI, myocardial infarction; CAD, coronary artery disease; SNP, single nucleotide polymorphism.

Cell types	Observed SNPs	Expected SNPs	Fold	*P*	Corrected *P*
Brain substantia nigra	10	1.3	7.7	1.00×10^−6^	8.10×10^−5^
Brain angular gyrus	10	1.7	6.1	4.00×10^−6^	3.24×10^−4^
Adipose nuclei	8	1.3	6.2	3.80×10^−5^	3.08×10^−3^
Induced pluripotent stem DF 19.11 cell line	7	1	7.2	4.80×10^−5^	3.89×10^−3^
Spleen	8	1.4	5.7	6.80×10^−5^	5.51×10^−3^
Brain anterior caudate	8	1.5	5.3	1.08×10^−4^	8.75×10^−3^
Brain cingulate gyrus	8	1.5	5.3	1.14×10^−4^	9.23×10^−3^
Embryonic stem cell line	4	0.3	11.9	3.67×10^−4^	2.97×10^−2^
Mobilized CD34 primary cells	5	0.6	8.1	3.78×10^−4^	3.06×10^−2^
Brain mid frontal lobe	7	1.5	4.8	5.64×10^−4^	4.57×10^−2^
Gastric	6	1	5.8	5.71×10^−4^	4.63×10^−2^

### Connectivity in a protein-protein interaction network among MI/CAD-associated SNPs

Finally, to investigate whether specific biological networks are expressed in specific cell types, we examined connectivity of protein-protein interaction (PPI) networks among the 45 GWAS loci (45 GWAS SNPs, as well as SNPs in *r* ^2^≥0.8) in different cell lines. Consistent with the HaploReg v2 and polygenic association analysis, we observed high direct connectivity in a PPI network involving known lipid genes, particularly apolipoprotein E (*APOE*), apolipoprotein C3 (*APOC3*) and low-density lipoprotein receptor (*LDLR*) in adipose nuclei (*P* = 0.002) and mesenchymal stem cell line derived adipocyte cultured cells (*P* = 0.001), and apolipoprotein B (*APOB*) and Sortilin 1 (*SORT1*) in adult liver (*P* = 0.009) (S11 Fig). Specific effects were observed in PPI networks in other cell types as well, including adult liver (S6 Table).

## Discussion

We utilized several complementary human genetic approaches to partition the genetic risk of MI/CAD into different genomic categories and cell types. Three principal findings emerged: (1) genetic variants residing in noncoding regions flanking protein-coding genes make up a large proportion of the heritability for MI/CAD; (2) association signals are enriched in histone modification marks; and (3) clear cell-type specific effects emerged with genetic effects of MI/CAD-associated SNPs being enhanced in adipocyte cell lines.

We highlight an important role for genetic variants residing within specific regulatory elements including promoters and enhancer regions, suggesting that the genetic risk for MI/CAD is largely driven by variants in key regulatory elements. Because we did not adjust for traditional risk factors of MI/CAD, our heritability estimates may include the fraction derived from these risk factors. Furthermore, our heritability estimate of 0.43 for the total genome is consistent with previous family-based studies with estimates of 0.3–0.63 [1,28,29]. Our work is consistent with previous studies that showed that a significantly higher portion of GWAS SNPs overlap regulatory elements such as transcription factor binding regions and/or DNase I hypersensitive sites [18,30]. These findings have important implications for interpretation of GWAS signals, particularly for identifying causal variants. Our findings particularly highlight an important role for promoter and/or enhancer regions in fine-mapping GWAS signals.

Our analyses further highlighted the genetic effects of MI/CAD-associated SNPs occurring in specific cell types, including mesenchymal and adipocyte cell lines. Adipocytes may affect cardiovascular homeostasis by regulating diverse peptides and nonpeptide compounds, which have been implicated in cardiovascular disease pathogenicity [31]. Furthermore, obesity is a casual risk factor for MI/CAD [32]. Here, we demonstrate that alteration of chromatin-mediated gene regulation by DNA sequence variation at key genomic regions within adipocytes is an important determinant of MI/CAD risk. Our results are consistent with studies that have shown that adipocytes play an important role in the pathogenesis of obesity, with adverse effects on inflammation, hemodynamics, and cardiovascular function [33]. We propose that the adipose-cardiovascular axis is not only an acquired mediator of MI/CAD risk but also a critical genetic determinant of MI/CAD risk in the general population. Furthermore, we highlight other loci that are not known to be involved in cardio-metabolism but appear to map to histone marks in the adipocyte cell lines. For example, SNPs in the *MIA3* and *MRAS* loci are highly associated with CAD [2–4] but have not been found to be associated with any cardio-metabolic intermediate trait [34]. Notably, we observe enrichment of MI/CAD-associated SNPs disrupting regulation in brain and spleen tissue raising new hypotheses in the pathogenesis of human atherosclerosis. Hypothalamic-pituitary regulation of the autonomic nervous system has a diverse array of impacts on cardiovascular disease determinants including blood pressure, heart rate, sodium regulation, metabolism, and physiologic responses to stress [35]. Furthermore, proinflammatory mediators within the spleen may have an important role in MI [36]. Because the spleen consists of a multitude of cell types, including immune B/T cells, macrophages, monocytes, endothelial cells, smooth muscle cells from larger arterioles, chromatin data of specific cell types in the spleen may be useful to identify specific functional roles related to MI. The mechanism by which these tissues contribute to a higher enrichment to MI/CAD associated SNPs may be through tissue-specific regulatory perturbation in functional regulatory regions (histone marks and/or chromatin enhancer states) adjacent to protein-coding regions, specifically in adipose, heart, brain and spleen tissues. Thus we propose that genetic determinants of early-onset myocardial infarction have biologic roles within distinct tissue types and these observations should prioritize experimental modeling strategies.

We note the following limitations. We note that the case samples in MIGen and WTCCC CAD were ascertained based on early-onset MI/CAD that was not fatal (male cases < 50 years old and female cases < 60 years old). Therefore, the genetic architecture and some of the tissue types highlighted in this study may be less relevant to the more general, broader CAD phenotype. Furthermore, we observed strong enrichment signals in some tissues (such as heart and intestinal mucosa) that may serve as a proxy for tissues that are biologically relevant to MI/CAD (such as smooth muscle in arterial walls). This may be due to tissues with similar cell types (i.e. smooth muscle cells in different tissues) having similar open chromatin structures. We note that we observed relatively weak signals for immune-related tissues and cell types despite a recent study suggesting that there was enrichment of tissue-specific eSNPs associated with CAD in pathways related to the immune system[37]. We have not examined our results in the context of biological pathways relevant to specific cell types (for example, immune/inflammation pathways in immune cells), and further investigation on this is warranted.

In summary, we have shown that disease-causing variants for MI/CAD are enriched in promoters and enhancer regions flanking protein-coding genes. Functional data from the NIH Roadmap Project and ENCODE provide key links to specific cell types such as mesenchymal stem cell derived adipocyte cultured cells, heart, brain and spleen cells with risk variants for MI/CAD. Our results highlight the importance of investigating the noncoding regions of the genome in genetic studies and suggest a key role of tissue-specific regulatory mechanisms on the etiology of MI/CAD. Identifying critical nodes that are significant drivers of a substantial portion of human disease can both inform biological investigation and prioritize therapeutic efforts.

## Materials and Methods

Details regarding material and methods for primary analyses presented in main figures and tables are provided below and in corresponding figure and table legends. Details about methods for supplemental figures and tables are provided in the legends in the Supporting Information.

### GWAS data for MI/CAD

We obtained GWAS data for 5,903 samples from MIGen [5] (2,905 cases and 2,998 controls) and 4,837 samples from WTCCC [38] (1,914 cases and 2,923 controls). For quality control, samples with extreme heterozygosity, gender mismatch or sample call rate <95% and SNPs with Hardy-Weinberg equilibrium *P*<10^−6^, minor allele frequency <1% or SNP call rate <98% were excluded prior to imputation.

### Imputation into the 1000 Genomes reference panel

We prephased MIGen GWAS data using the MaCH software and imputed into the 1000 Genomes Phase I Integrated Release Version 3 Haplotypes panel using minimac [39]. We imputed WTCCC CAD data using IMPUTE2 [39–41]. To control for imputation quality, we removed low-frequency SNPs with minor allele frequency <0.5% and used SNPs with high imputation quality metric (minimac rsq or IMPUTE2 info) >0.5. We tested for association with additive tests for imputed dosage data using the SNPTEST [40,42] and PLINK [43] software. Gender, age and principal components for population structure [44] were used for covariates in the association analysis.

### Genomic annotation

A categorization of SNPs was adopted in order to compare their relative aggregate properties (S1 Table). “Genic” regions were defined as ±10 kb of the 3′ or 5′ untranslated regions (UTR) of a gene. “Intergenic” regions were those that were beyond ±10 kb of the 3′ or 5′ UTR of a gene. “Genic coding” variants were those that code amino acid sequence (nonsense, missense, synonymous). “Genic noncoding” variants were those that were resided outside of the coding region but within the “genic” window size. This includes the region beyond the 3’ or 5’ UTR, as well as the introns. 19.5% of variants in the “genic noncoding” region are also observed in the DHS regions as defined by Gusev et al [45]. Genomic compartments for the “genic coding”, “genic noncoding”, and “intergenic” regions were defined to be non-overlapping. Other window sizes for “genic” regions of ±20 kb and ±50 kb were also tested.

### Regulatory element annotation

To examine polygenic effects of specific chromatin marks, we obtained histone modification marks (histone H3 lysine 4 trimethylation or H3K4me3, histone H3 lysine 9 acetylation or H3K9ac, histone H3 lysine 27 acetylation or H3K27ac) in diverse cell types or tissues that are indicative of active enhancers or promoters from the NIH Roadmap Epigenomics Project [14,15] data repository (see **URL**). We identified histone mark using the MACS v1.4 software [46], with a *P* value cutoff of 10^−5^ and a false discovery rate cutoff of 0.01. We ran the histone mark test files with control files matched by individual if available in order to increase specificity. We also obtained chromatin core 15-state data for 16 ENCODE cell lines (Epigenome ID E114-E119) (see **URL**). We examined eight active chromatin states (active transcription start site [TSS] proximal promoter states [active TSS, flanking active TSS], a transcribed state at the 5′ and 3′ end of genes showing both promoter and enhancer signatures [transcription at gene 5′ and 3′], actively transcribed states [strong transcription, weak transcription], enhancer states [enhancers, genic enhancers], and a state associated with zinc finger protein genes [ZNF and repeats]) and seven inactive states (constitutive heterochromatin, bivalent regulatory states [bivalent TSS, flanking bivalent TSS/enhancers, bivalent enhancers], repressed PolyComb states [repressed PolyComb, weak repressed PolyComb], and a quiescent state) inferred by ChromHMM [15].

### Polygenic risk score analysis across three distinct variant sets

Polygenic risk score (*PRS*_*i*_) for individual *i* for a given variant set was calculated as $PRSi\;=\;\sum_{j\in SNPs}\beta_{j}\times d_{ij}$, where *β*_*j*_ >0 is the natural log of odds ratio for the risk allele of SNP *j* from the association result in the MIGen discovery set and *d*_*ij*_ is the dosage (0−2) for that same allele of individual *i* from the WTCCC CAD validation set, as previously described [12]. Association of polygenic risk scores with disease status was tested using the Wald test and variance explained was estimated by Nagelkerke’s *R* ^2^ from logistic regression [47]. Variance explained after normalization was calculated using Nagelkerke’s *R* ^2^ divided by the number of SNPs. We used different discovery *P* value thresholds in MIGen (association *P*<5×10^−8^, 5×10^−7^, 5×10^−6^, 5×10^−5^, 5×10^−4^, 0.005, 0.05, 0.1, 0.2, 0.3, 0.4, 0.5, 1) to define various polygenic risk scores (Fig 1).

### SNP-heritability analysis across three distinct variant sets

The proportion of phenotypic variance explained by each variant set was estimated using the restricted maximum likelihood method [48] implemented in the Genome-wide Complex Trait Analysis (GCTA) software, transformed to the underlying liability scale assuming a prevalence of 6% for CAD [22,23]. We removed one of each pair of individuals with estimated relatedness larger than 0.05 (grm-cutoff 0.05 in the GCTA software). Given a previous observation that LD does not substantially influence polygenic risk score analysis [49] or heritability explained by genotyped SNPs [13], and that imputed SNPs do not produce biased heritability estimates compared to genotyped SNPs [13], we included all SNPs in our variant sets (Table 1). We performed heritability estimates independently in the MIGen and WTCCC CAD studies, and then a meta-analysis across both studies using as weights the inverse variance (Tables 1 and S2–S4). We also estimated heritability with a prevalence of 3% for early-onset MI/CAD (S5 Table).

### Polygenic risk score, enrichment and heritability analysis of three histone modification marks across cell types

We performed analyses on SNPs within three histone marks (H3K27ac, H3K4me3, H3K9ac) in different cell types. We performed polygenic risk score analysis as described above. We used a discovery *P* value threshold of <0.05 in MIGen to form the polygenic risk score and then validation in WTCCC CAD (Fig 2A). We performed enrichment analysis by comparing the proportion of significant variants passing a specific association *P* threshold of a variant set with that of a baseline set. We tested different association *P* thresholds *P*<5×10^−7^, 5×10^−6^, 5×10^−5^. For the baseline set, we examined variants that resided in the intergenic regions, were not conserved (Genomic Evolutionary Rate Profiling score [50] <5) and did not overlap with any of the studied regulatory elements (Fig 2B). We performed heritability analysis as described above. We restricted heritability analysis to variants within histone marks for the indicated cell line using the restricted maximum likelihood method [48] implemented in the GCTA software (Fig 2C) [22,23]. For all approaches, we performed analyses restricting to SNPs residing in the three histone marks (H3K27ac, H3K4me3, H3K9ac) that were present in the different cell types

### Enrichment analysis of MI/CAD-associated SNPs in specific cell types

For enrichment analyses, we selected 45 independent SNPs that were shown to be significantly associated with CAD in a large-scale GWAS meta-analysis (Table 2) [8]. We also included tag SNPs in strong LD (*r* ^2^≥0.8 in 379 individuals from European populations [85 CEU, 98 TSI, 93 FIN, 89 GBR, 14 IBS] from the 1000 Genomes Project [20]) with the 45 GWAS SNPs. We utilized the NIH Roadmap and ENCODE data and two mammalian conservation algorithms, GERP and SiPhy-omega, implemented in HaploReg v2 [26] (see **URL**) to examine if the 45 GWAS loci are enriched in regions of inferred strong enhancer chromatin states [27] in specific cell types. The background set for enhancer enrichment analysis was “All SNPs in 1000 Genomes phase I data”.

### Hierarchical clustering of MI/CAD-associated SNPs, histone modification marks and specific cell types

For hierarchical clustering analysis, we mapped all 45 MI/CAD GWAS SNPs, along with SNPs in high LD (*r* ^2^≥0.8) (same SNP set in enrichment analysis), to each of the three histone marks (H3K27ac, H3K4me3 and H3K9ac), which are associated with active regulatory regions, in different cell types (Fig 3). Hierarchical clustering was performed using the heatmap function in R (R Project for Statistical Computing). Reordering of the rows and columns to produce the dendrogram was based on the presence or absence of a SNP residing in a histone mark in different cell types.

### Protein connectivity of MI/CAD-associated SNPs in specific cell types

We tested for direct connectivity of genes in GWAS loci in specific cell types. We tested 45 MI/CAD GWAS SNPs, in addition to SNPs in high LD (*r* ^2^≥0.8) (same SNP set in enrichment analysis), that overlapped with the three histone marks (H3K4me3, H3K9ac, H3K27ac) in a specific cell type. DAPPLE [51] was utilized to test for direct connectivity in PPI networks. Gene regulatory regions were defined as within 110 kb upstream of transcription start site and 40 kb downstream of transcription end site of each of the 45 lead SNPs or tag SNPs were included in the analysis. We performed 1,000 permutations to obtain empirical significance for the observed connectivity compared with the expected connectivity.

### Web resources

NIH Roadmap Epigenomics Project, http://www.roadmapepigenomics.org ENCODE Chromatin Core 15-State data, http://egg2.wustl.edu/roadmap/data/byFileType/chromhmmSegmentations/ChmmModels/coreMarks/jointModel/final/ HaploReg v2, http://www.broadinstitute.org/mammals/haploreg/haploreg.php

## Supporting Information

S1 Fig Explained variability using Nagelkerke’s *R* ^2^ from polygenic risk score analysis of select genomic compartments (10 kilobases window for genic regions). Polygenic risk score analysis was performed across three different genomic compartments. The top bar plot represents the explained variability using Nagelkerke’s *R* ^2^ of the logistic regression models for the polygenic risk score analysis whereas the bottom bar plot represents the number of SNPs within each of the compartments. The strongest signals for explained variability were within noncoding regions adjacent to protein-coding genes (“genic noncoding”). SNP, single nucleotide polymorphism. Genic coding, variants that code amino acid sequence within ±10 kilobases of the 3′ or 5′ untranslated regions of a gene. Genic noncoding, variants that do not code amino acid sequence within ±10 kilobases of the 3′ or 5′ untranslated regions of a gene. Intergenic variants that are beyond ±10 kilobases of the 3′ or 5′ untranslated regions of a gene.
(PDF)

S2 Fig Contributions of three genomic compartments to the polygenic risk of MI/CAD and explained variability using Nagelkerke’s *R* ^2^ from polygenic risk score analysis of select genomic compartments (20 kilobases window for genic regions). Polygenic risk score analysis was performed across three different genomic compartments. The top bar plot represents the explained variability using Nagelkerke’s *R* ^2^ of the logistic regression models for the polygenic risk score analysis whereas the bottom bar plot represents the number of SNPs within each of the compartments. The strongest signals for explained variability were within noncoding regions adjacent to protein-coding genes (“genic noncoding”). SNP, single nucleotide polymorphism. Genic coding, variants that code amino acid sequence within ±20 kilobases of the 3′ or 5′ untranslated regions of a gene. Genic noncoding, variants that do not code amino acid sequence within ±20 kilobases of the 3′ or 5′ untranslated regions of a gene. Intergenic variants that are beyond ±20 kilobases of the 3′ or 5′ untranslated regions of a gene.
(PDF)

S3 Fig Contributions of three genomic compartments to the polygenic risk score of MI/CAD and explained variability using Nagelkerke’s *R* ^2^ from polygenic risk score analysis of select genomic compartments (50 kilobases window for genic regions). Polygenic risk score analysis was performed across three different genomic compartments. The top bar plot represents the explained variability using Nagelkerke’s *R* ^2^ of the logistic regression models for the polygenic risk score analysis whereas the bottom bar plot represents the number of SNPs within each of the compartments. The strongest signals for explained variability were within noncoding regions adjacent to protein-coding genes (“genic noncoding”). SNP, single nucleotide polymorphism. Genic coding, variants that code amino acid sequence within ±50 kilobases of the 3′ or 5′ untranslated regions of a gene. Genic noncoding, variants that do not code amino acid sequence within ±50 kilobases of the 3′ or 5′ untranslated regions of a gene. Intergenic variants that are beyond ±50 kilobases of the 3′ or 5′ untranslated regions of a gene.
(PDF)

S4 Fig Polygenic risk score analysis of select genomic compartments after excluding 45 GWAS loci for MI/CAD. Polygenic risk score analysis was performed across three different genomic compartments. The top bar plot represents the strength of association for the polygenic risk score analysis whereas the bottom bar plot represents the number of SNPs within each of the compartments. The strongest polygenic association signals were within noncoding regions adjacent to protein-coding genes (“genic noncoding”). GWAS, genome-wide association study; MI, myocardial infarction; CAD, coronary artery disease; SNP, single nucleotide polymorphism. Genic coding, variants that code amino acid sequence within ±10 kilobases of the 3′ or 5′ untranslated regions of a gene. Genic noncoding, variants that do not code amino acid sequence within ±10 kilobases of the 3′ or 5′ untranslated regions of a gene. Intergenic variants that are beyond ±10 kilobases of the 3′ or 5′ untranslated regions of a gene.
(PDF)

S5 Fig Strength of polygenic association signal within regulatory elements. For analyses of polygenic association signal within regulatory elements, we constructed a polygenic risk score comprised of SNPs within each of the three histone modification marks (H3K27ac, H3K4me3 and H3K9ac) with *P*<0.05 in the MIGen discovery set. We tested for association of this polygenic risk score in the WTCCC CAD validation set. As a baseline control set, we also test SNPs in regions that are outside of these histone marks within 10 kilobases (kb) of the protein coding regions of the genome. To reduce the effects of linkage disequilibrium, these baseline SNPs were selected to be 5 kb away from the histone marks. We observed that this signal was stronger in these histone marks beyond what we expect by chance after randomly sampling “genic noncoding” regions outside of the marks (Mann-Whitney test *P* = 1.1×10^−95^). CAD, coronary artery disease; SNP, single nucleotide polymorphism.
(PDF)

S6 Fig Polygenic and heritability analysis of eight active chromatin states across cell types in the ENCODE data. We performed polygenic risk score and heritability analyses to test for cell type specific effects on the genetic risk for MI/CAD. Analyses were conducted on SNPs residing in eight active chromatin states inferred by ChromHMM [15] that were present in the different cell types. (A) Polygenic risk score analysis. We performed polygenic risk score association analysis on SNPs with MIGen discovery association *P*<0.05. Negative logarithm of *P* values from association testing of the polygenic risk score performed in the WTCCC CAD was shown. Cell types were sorted based on the strength of polygenic association. Orange vertical line represents a significant level with 5% alpha error. (B) Heritability analysis. Heritability analysis was performed within chromatin states in the MIGen study. Each point in the plot represents the variance in liability generated from a joint model involving two variance components using the Genome-wide Complex Trait Analysis software [22,23]. The two variance components include 1) SNPs in the specified chromatin state that was present in the indicated cell type and 2) all other SNPs outside of these regions. The variance in liability is an estimate from the ratio of genetic variance to phenotypic variance for the specified variance component (i.e. the specified variance component is all SNPs within the specified chromatin state) whereas the *P* value is from the likelihood ratio test of a reduce model with the specified genetic variance component dropped from the full model, from the restricted maximum likelihood method in the Genome-wide Complex Trait Analysis software [22,23]. MI, myocardial infarction; CAD, coronary artery disease; SNP, single nucleotide polymorphism.
(PDF)

S7 Fig Polygenic and heritability analysis of seven inactive chromatin states across cell types in the ENCODE data. We performed polygenic risk score and heritability analyses to test for cell type specific effects on the genetic risk for MI/CAD. Analyses were conducted on SNPs residing in seven inactive chromatin states inferred by ChromHMM [15] that were present in the different cell types. (A) Polygenic risk score analysis. We performed polygenic risk score association analysis on SNPs with MIGen discovery association *P*<0.05. Negative logarithm of *P* values from association testing of the polygenic risk score performed in the WTCCC CAD was shown. Cell types were sorted based on the strength of polygenic association. Orange vertical line represents a significant level with 5% alpha error. (B) Heritability analysis. Heritability analysis was performed within chromatin states in the MIGen study. Each point in the plot represents the variance in liability generated from a joint model involving two variance components using the Genome-wide Complex Trait Analysis software [22,23]. The two variance components include 1) SNPs in the specified chromatin state that was present in the indicated cell type and 2) all other SNPs outside of these regions. The variance in liability is an estimate from the ratio of genetic variance to phenotypic variance for the specified variance component (i.e. the specified variance component is all SNPs within the specified chromatin state) whereas the *P* value is from the likelihood ratio test of a reduce model with the specified genetic variance component dropped from the full model, from the restricted maximum likelihood method in the Genome-wide Complex Trait Analysis software [22,23]. MI, myocardial infarction; CAD, coronary artery disease; SNP, single nucleotide polymorphism.
(PDF)

S8 Fig Disproportionate number of MI/CAD GWAS SNPs in regulatory elements. We examined overlap of 45 MI/CAD GWAS SNPs in three histone marks. We generated random sets to determine statistical significance of this overlap. We excluded two SNPs out of the 45 GWAS SNPs because we were unable to find appropriate matching null SNPs (rs3798220 and rs12205331). Histogram is drawn based on 10,000 permutations of random sets of 43 variants from the SNPsnap software [25]. A median of 15 of 43 random SNPs overlap any of three histone marks across diverse cell types and tissues. Random SNPs were selected to match the query GWAS SNPs based on similar minor allele frequency (±0.05 frequency), number of SNPs in LD with query SNP (±10% of number of SNPs in LD with query SNP using *r* ^2^>0.5), distance to nearest gene (±10% of distance of nearest gene from query SNPs) and gene density (±10% of number of genes in loci around the query SNPs) [25]. Compared to the random variant sets, we observed a statistically significant higher number of GWAS SNPs (25 out of 43) overlapping the histone marks (*P* = 4×10^−4^). MI, myocardial infarction; CAD, coronary artery disease; GWAS, genome-wide association study; SNP, single nucleotide polymorphism; LD, linkage disequilibrium.
(PDF)

S9 Fig Hierarchical clustering of 45 MI/CAD GWAS SNPs and specific cell types for a histone modification mark (H3K9ac). We mapped 45 MI/CAD GWAS SNPs, as well as SNPs in high linkage disequilibrium (*r* ^2^≥0.8), to H3K9ac in different cell types. Hierarchical clustering was based on the presence or absence of a SNP residing in H3K9ac in different cell types and was performed using the heatmap function in the R Project for Statistical Computing. We observed unique patterns between the different GWAS loci and cell types. MI, myocardial infarction; CAD, coronary artery disease; GWAS, genome-wide association study; SNP, single nucleotide polymorphism.
(PDF)

S10 Fig Hierarchical clustering of 45 MI/CAD GWAS SNPs and specific cell types for a histone modification mark (H3K4me3). We mapped 45 MI/CAD GWAS SNPs, as well as SNPs in high linkage disequilibrium (*r* ^2^≥0.8), to H3K4me3 in different cell types. Hierarchical clustering was based on the presence or absence of a SNP residing in H3K4me3 in different cell types and was performed using the heatmap function in the R Project for Statistical Computing. We observed unique patterns between the different GWAS loci and cell types. MI, myocardial infarction; CAD, coronary artery disease; GWAS, genome-wide association study; SNP, single nucleotide polymorphism.
(PDF)

S11 Fig High connectivity in a protein-protein interaction network among *APOE*, *APOC3*, and *LDLR*. We tested for direct connectivity of genes in GWAS loci in specific cell types. We tested 45 MI/CAD GWAS SNPs, in addition to SNPs in high linkage disequilibrium (*r* ^2^≥0.8) (same SNP set in enrichment analysis), that overlapped with the three histone marks (H3K4me3, H3K9ac, H3K27ac) in a specific cell type. SNPs that only overlap the three histone marks in adipose nuclei and mesenchymal stem cell (MSC) derived adipocyte cell types were tested. DAPPLE [51] was utilized to test for direct connectivity in protein-protein interaction (PPI) networks. Gene regulatory regions were defined as within 110 kb upstream of transcription start site and 40 kb downstream of transcription end site of each of the 45 lead SNPs or tag SNPs were included in the analysis. We tested each variant set 1,000 times to obtain empirical significance for the observed connectivity compared with the expected connectivity. We observed high direct connectivity in a PPI network comprised of known lipid genes (for example, apolipoprotein E [*APOE*], apolipoprotein C3 [*APOC3*], low-density lipoprotein receptor [*LDLR*]) in adipose nuclei and MSC derived adipocyte cell types.
(PDF)

S1 Table Functional categorization of variants. (DOCX)

S2 Table Heritability of MI/CAD explained by three genomic compartment sets (10 kilobases window for genic regions). (DOCX)

S3 Table Heritability of MI/CAD explained by three genomic compartment sets (20 kilobases window for genic regions). (DOCX)

S4 Table Heritability of MI/CAD explained by three genomic compartment sets (50 kilobases window for genic regions). (DOCX)

S5 Table Heritability with a prevalence of 3% of MI/CAD explained by three genomic compartment sets. (DOCX)

S6 Table Cell-type specific protein-protein interaction network among neighboring genes of 45 MI/CAD GWAS loci on three histone marks. (DOCX)
