# Supplementary figures and images for: Disproportionate Contributions of Select Genomic Compartments and Cell Types to Genetic Risk for Coronary Artery Disease
Source: PLoS Genet. 2015 Oct 28;11(10):e1005622. doi: 10.1371/journal.pgen.1005622 (PMC4625039; doi:10.1371/journal.pgen.1005622)

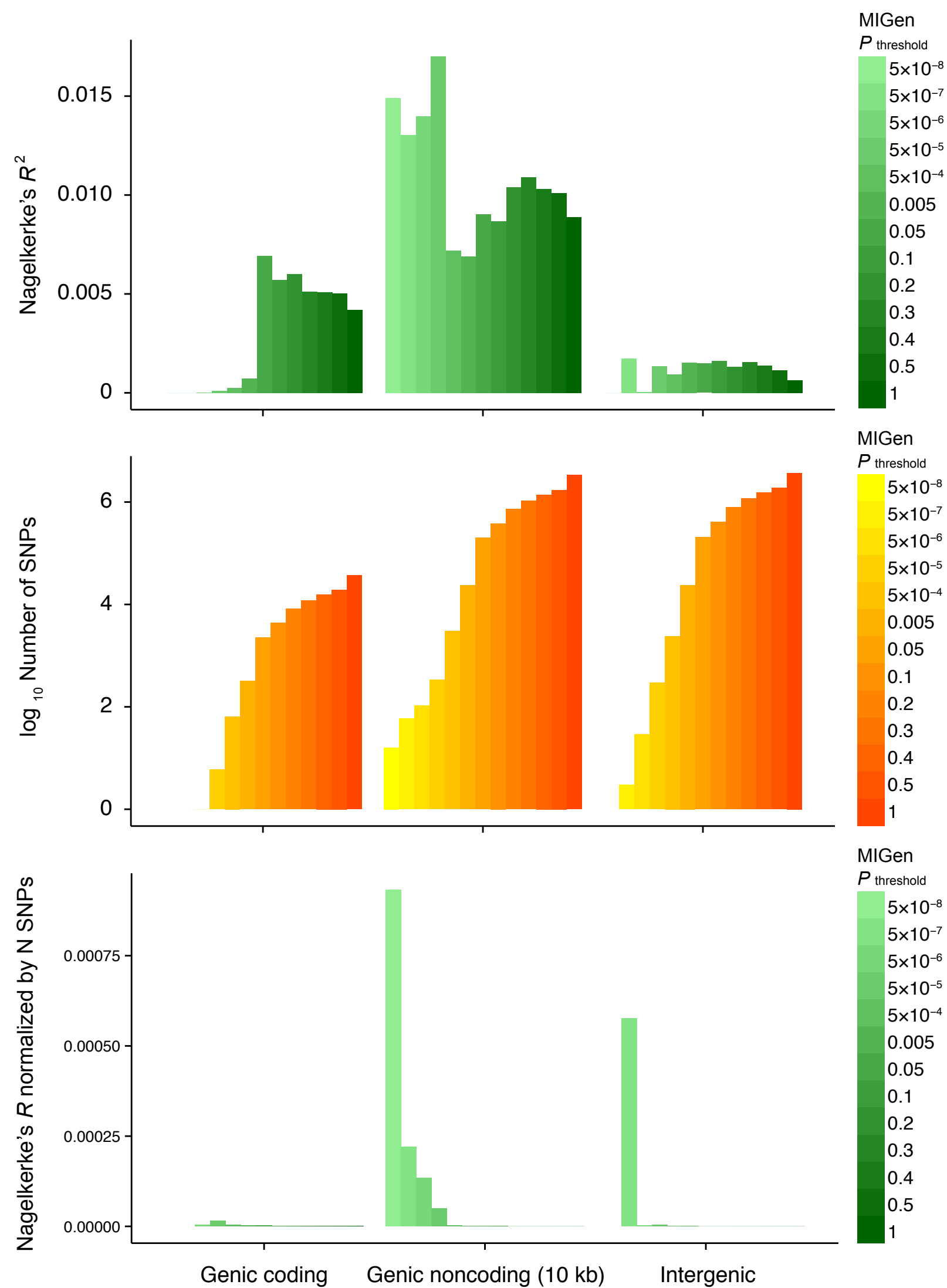

Supplement: S1 Fig — Polygenic risk score analysis was performed across three different genomic compartments. The top bar plot represents the explained variability using Nagelkerke’s R 2 of the logistic regression models for the polygenic risk score analysis whereas the bottom bar plot represents the number of SNPs within each of the compartments. The strongest signals for explained variability were within noncoding regions adjacent to protein-coding genes (“genic noncoding”). SNP, single nucleotide polymorphism. Genic coding, variants that code amino acid sequence within ±10 kilobases of the 3′ or 5′ untranslated regions of a gene. Genic noncoding, variants that do not code amino acid sequence within ±10 kilobases of the 3′ or 5′ untranslated regions of a gene. Intergenic variants that are beyond ±10 kilobases of the 3′ or 5′ untranslated regions of a gene. (PDF) [file pgen.1005622.s001.pdf]

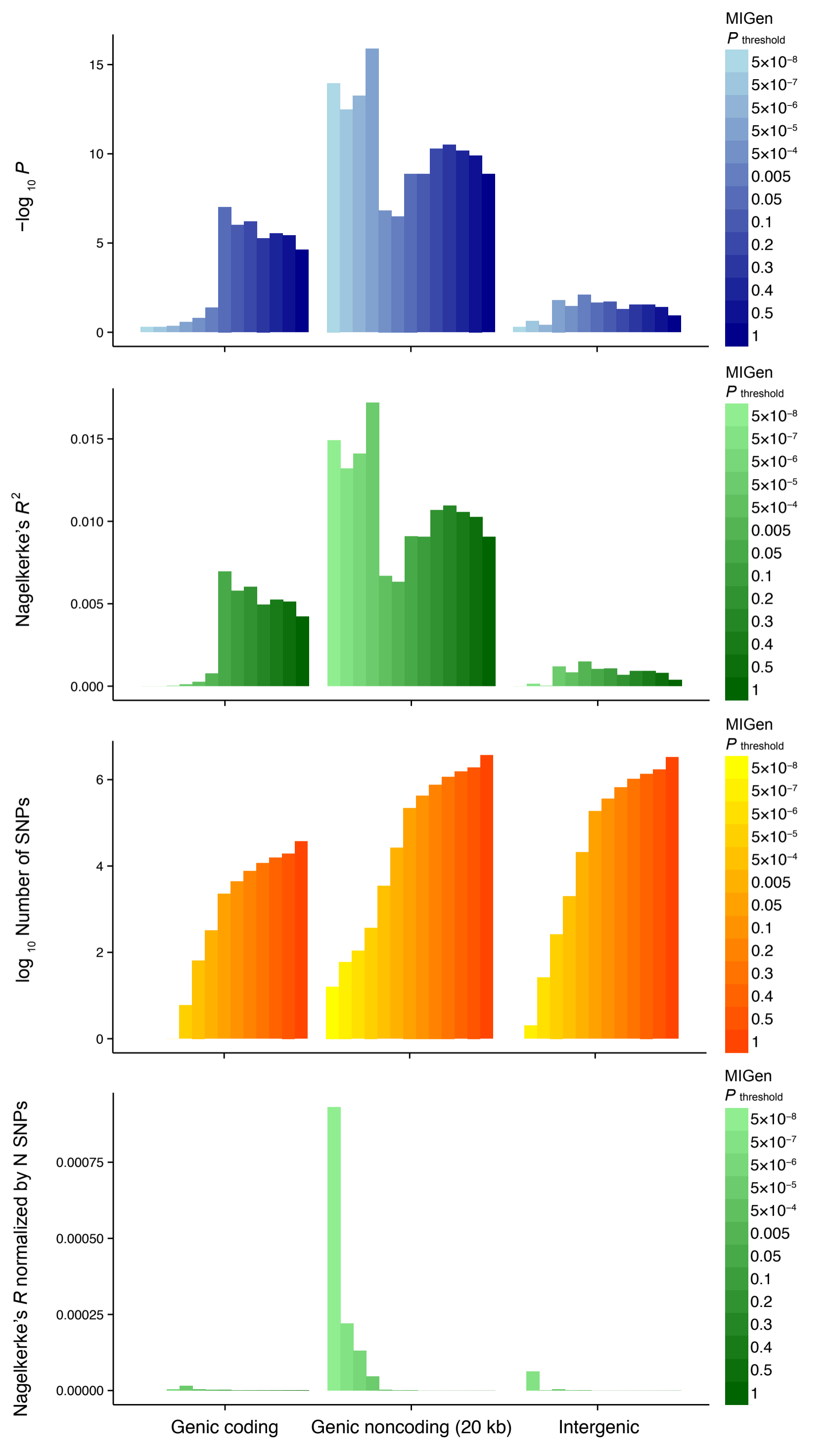

Supplement: S2 Fig — Polygenic risk score analysis was performed across three different genomic compartments. The top bar plot represents the explained variability using Nagelkerke’s R 2 of the logistic regression models for the polygenic risk score analysis whereas the bottom bar plot represents the number of SNPs within each of the compartments. The strongest signals for explained variability were within noncoding regions adjacent to protein-coding genes (“genic noncoding”). SNP, single nucleotide polymorphism. Genic coding, variants that code amino acid sequence within ±20 kilobases of the 3′ or 5′ untranslated regions of a gene. Genic noncoding, variants that do not code amino acid sequence within ±20 kilobases of the 3′ or 5′ untranslated regions of a gene. Intergenic variants that are beyond ±20 kilobases of the 3′ or 5′ untranslated regions of a gene. (PDF) [file pgen.1005622.s002.pdf]

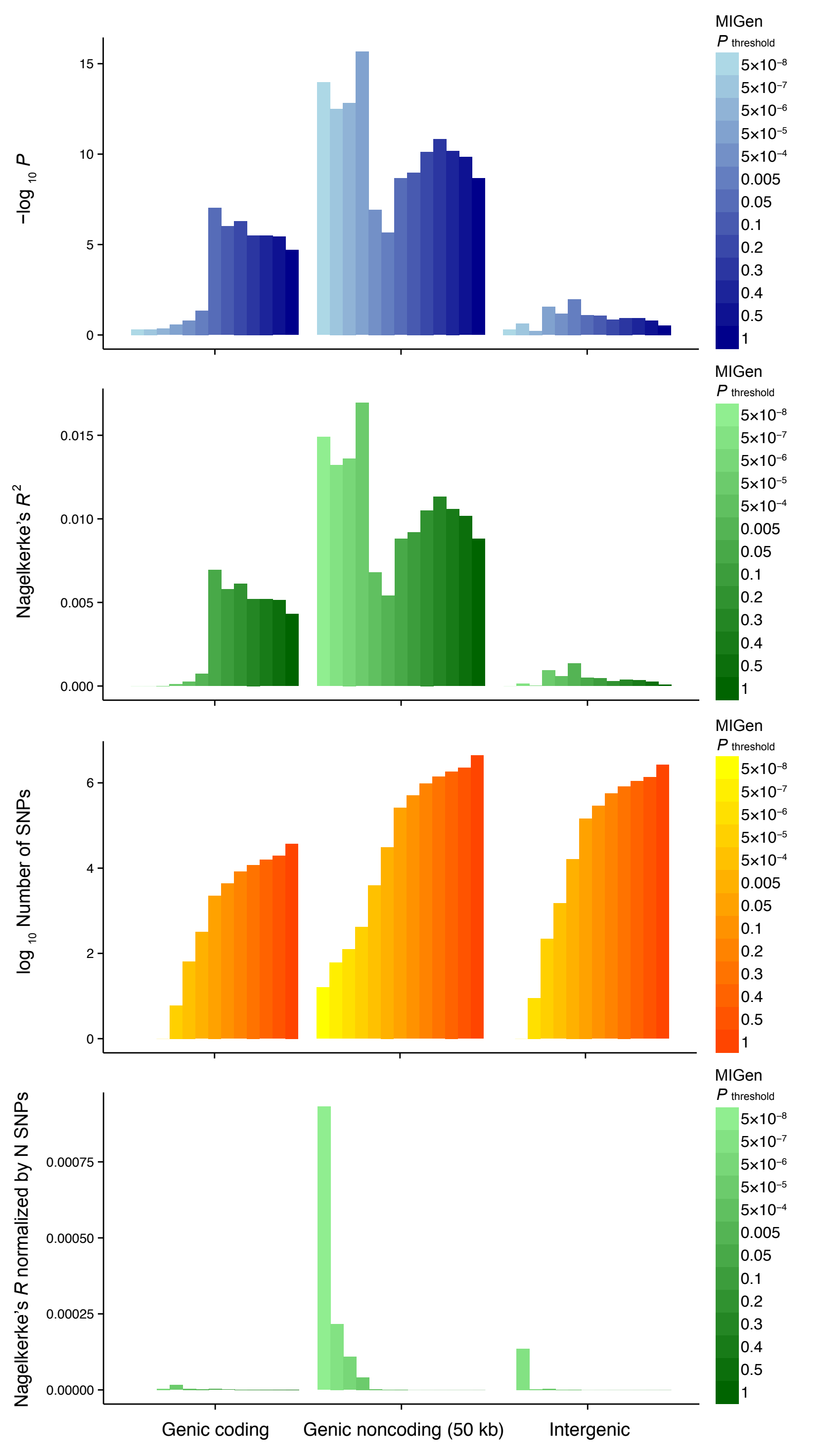

Supplement: S3 Fig — Polygenic risk score analysis was performed across three different genomic compartments. The top bar plot represents the explained variability using Nagelkerke’s R 2 of the logistic regression models for the polygenic risk score analysis whereas the bottom bar plot represents the number of SNPs within each of the compartments. The strongest signals for explained variability were within noncoding regions adjacent to protein-coding genes (“genic noncoding”). SNP, single nucleotide polymorphism. Genic coding, variants that code amino acid sequence within ±50 kilobases of the 3′ or 5′ untranslated regions of a gene. Genic noncoding, variants that do not code amino acid sequence within ±50 kilobases of the 3′ or 5′ untranslated regions of a gene. Intergenic variants that are beyond ±50 kilobases of the 3′ or 5′ untranslated regions of a gene. (PDF) [file pgen.1005622.s003.pdf]

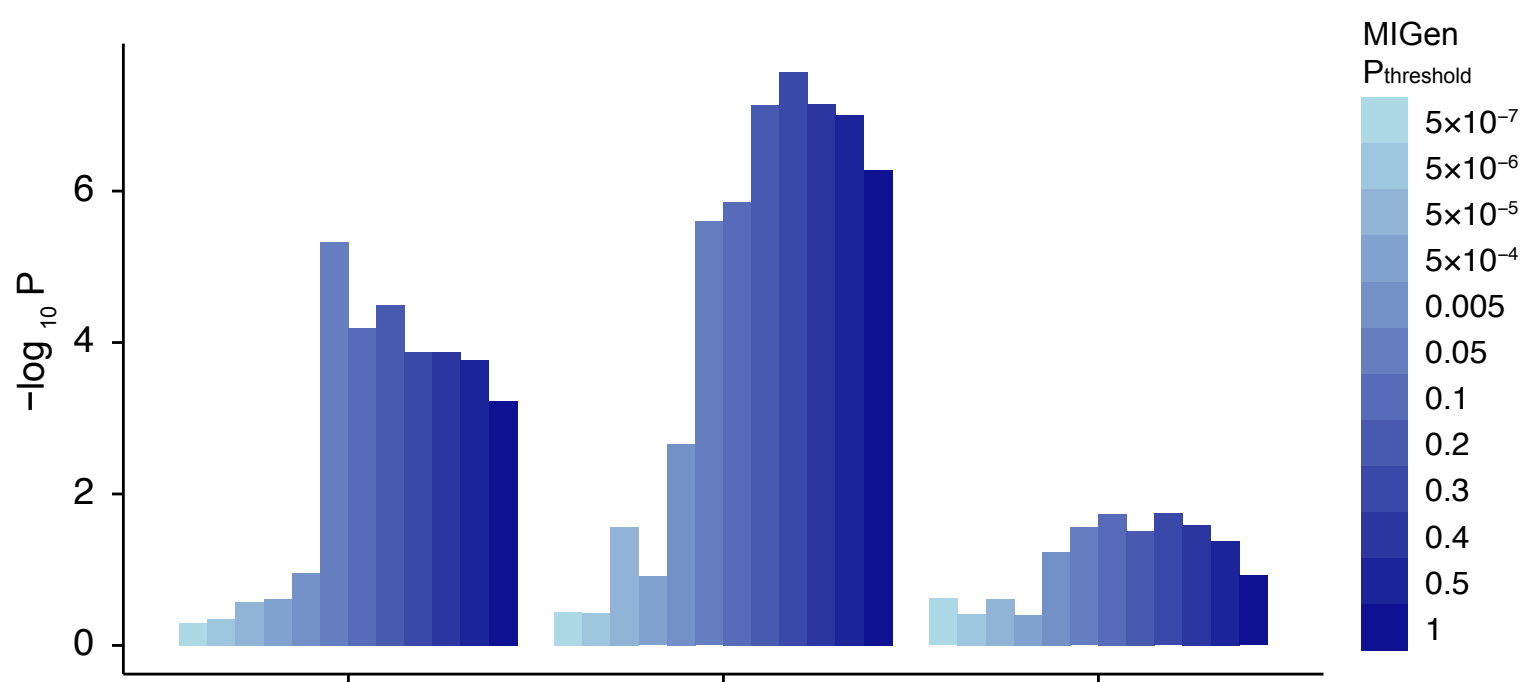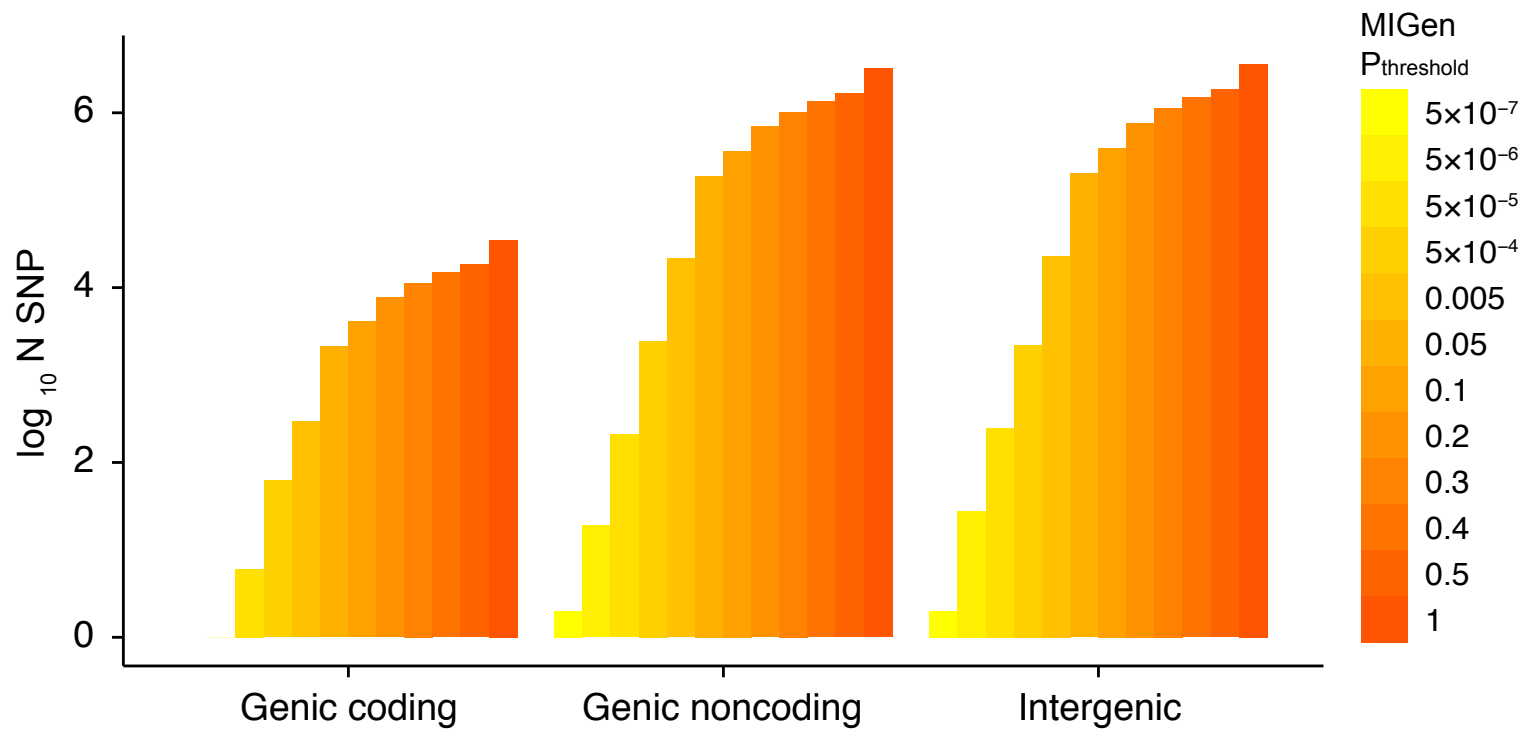

Supplement: S4 Fig — Polygenic risk score analysis was performed across three different genomic compartments. The top bar plot represents the strength of association for the polygenic risk score analysis whereas the bottom bar plot represents the number of SNPs within each of the compartments. The strongest polygenic association signals were within noncoding regions adjacent to protein-coding genes (“genic noncoding”). GWAS, genome-wide association study; MI, myocardial infarction; CAD, coronary artery disease; SNP, single nucleotide polymorphism. Genic coding, variants that code amino acid sequence within ±10 kilobases of the 3′ or 5′ untranslated regions of a gene. Genic noncoding, variants that do not code amino acid sequence within ±10 kilobases of the 3′ or 5′ untranslated regions of a gene. Intergenic variants that are beyond ±10 kilobases of the 3′ or 5′ untranslated regions of a gene. (PDF) [file pgen.1005622.s004.pdf]

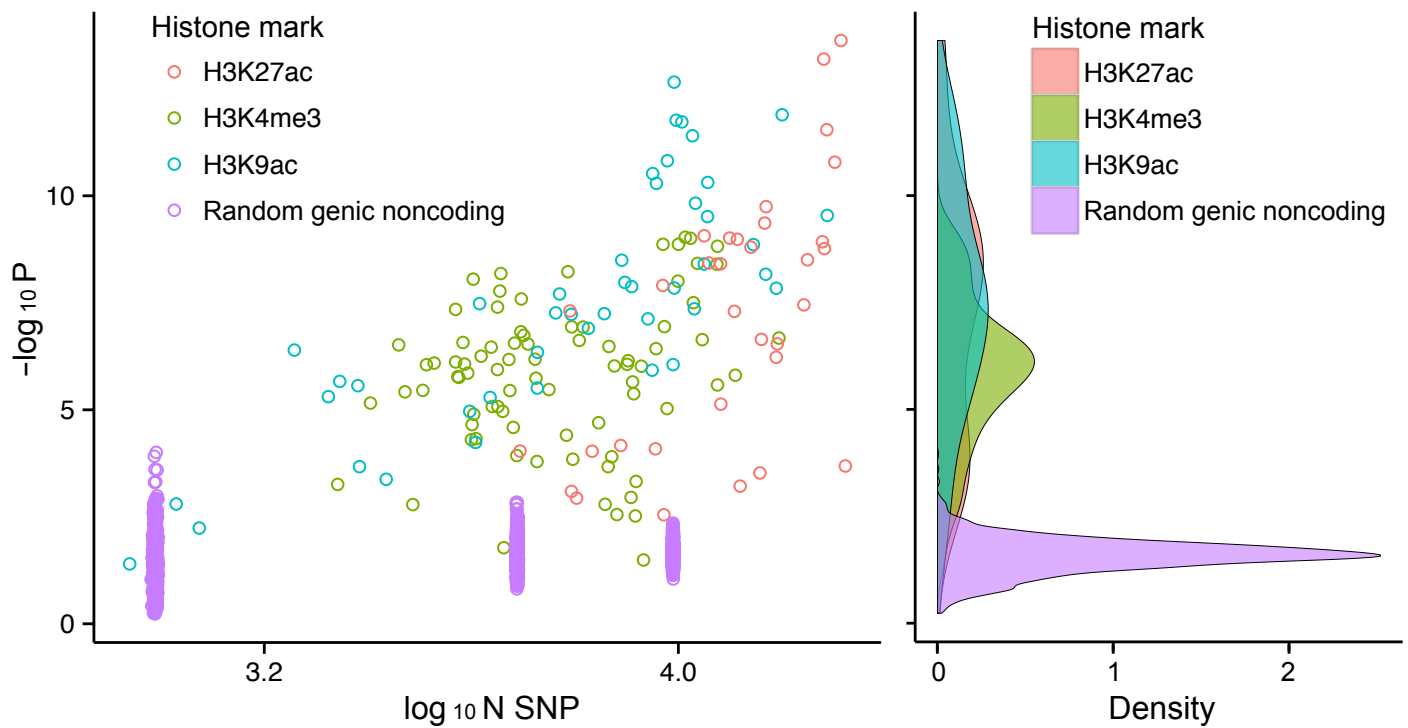

Supplement: S5 Fig — For analyses of polygenic association signal within regulatory elements, we constructed a polygenic risk score comprised of SNPs within each of the three histone modification marks (H3K27ac, H3K4me3 and H3K9ac) with P<0.05 in the MIGen discovery set. We tested for association of this polygenic risk score in the WTCCC CAD validation set. As a baseline control set, we also test SNPs in regions that are outside of these histone marks within 10 kilobases (kb) of the protein coding regions of the genome. To reduce the effects of linkage disequilibrium, these baseline SNPs were selected to be 5 kb away from the histone marks. We observed that this signal was stronger in these histone marks beyond what we expect by chance after randomly sampling “genic noncoding” regions outside of the marks (Mann-Whitney test P = 1.1×10^−95^). CAD, coronary artery disease; SNP, single nucleotide polymorphism. (PDF) [file pgen.1005622.s005.pdf]

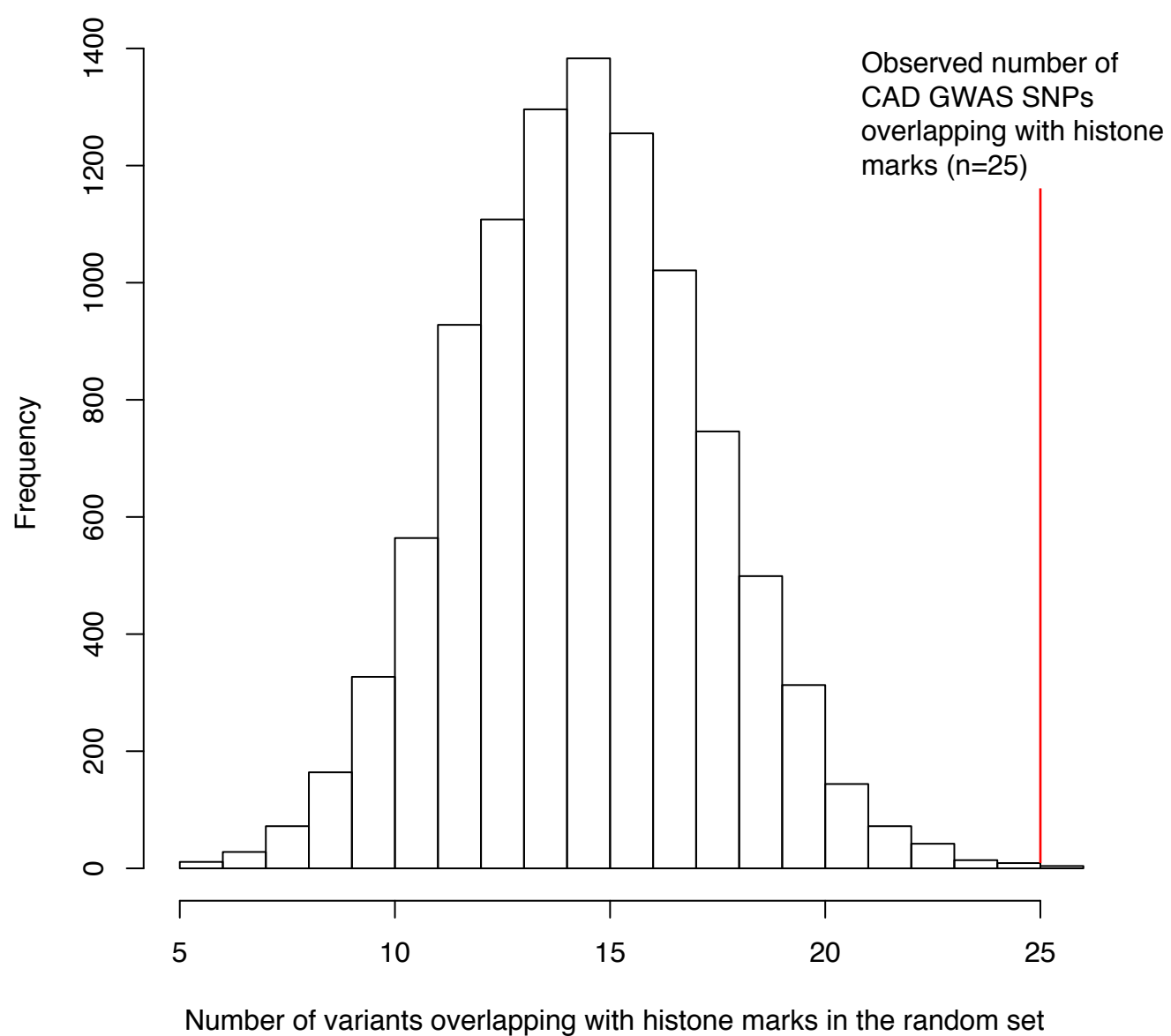

Supplement: S8 Fig — We examined overlap of 45 MI/CAD GWAS SNPs in three histone marks. We generated random sets to determine statistical significance of this overlap. We excluded two SNPs out of the 45 GWAS SNPs because we were unable to find appropriate matching null SNPs (rs3798220 and rs12205331). Histogram is drawn based on 10,000 permutations of random sets of 43 variants from the SNPsnap software [25]. A median of 15 of 43 random SNPs overlap any of three histone marks across diverse cell types and tissues. Random SNPs were selected to match the query GWAS SNPs based on similar minor allele frequency (±0.05 frequency), number of SNPs in LD with query SNP (±10% of number of SNPs in LD with query SNP using r 2>0.5), distance to nearest gene (±10% of distance of nearest gene from query SNPs) and gene density (±10% of number of genes in loci around the query SNPs) [25]. Compared to the random variant sets, we observed a statistically significant higher number of GWAS SNPs (25 out of 43) overlapping the histone marks (P = 4×10^−4^). MI, myocardial infarction; CAD, coronary artery disease; GWAS, genome-wide association study; SNP, single nucleotide polymorphism; LD, linkage disequilibrium. (PDF) [file pgen.1005622.s008.pdf]

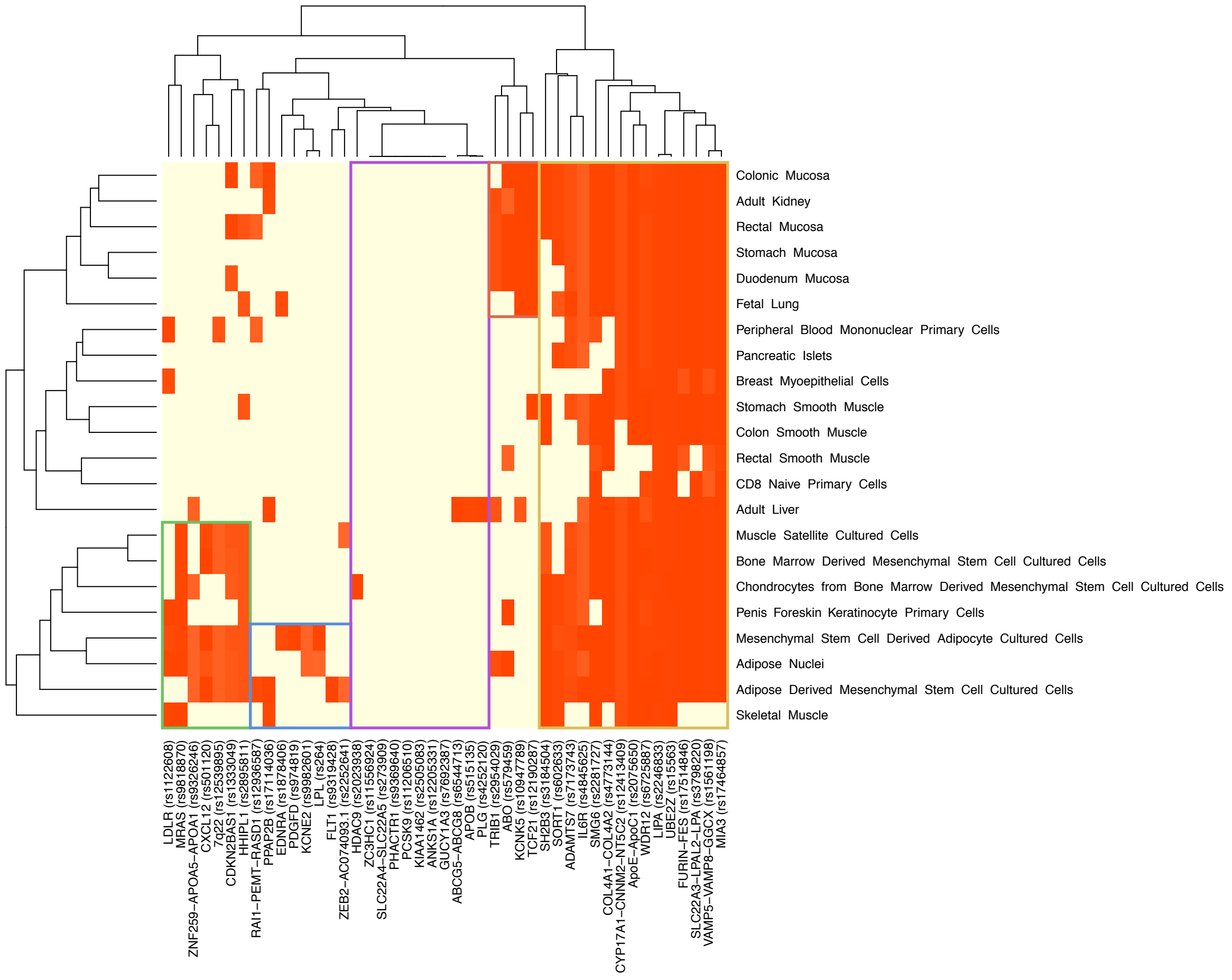

Supplement: S9 Fig — We mapped 45 MI/CAD GWAS SNPs, as well as SNPs in high linkage disequilibrium (r 2≥0.8), to H3K9ac in different cell types. Hierarchical clustering was based on the presence or absence of a SNP residing in H3K9ac in different cell types and was performed using the heatmap function in the R Project for Statistical Computing. We observed unique patterns between the different GWAS loci and cell types. MI, myocardial infarction; CAD, coronary artery disease; GWAS, genome-wide association study; SNP, single nucleotide polymorphism. (PDF) [file pgen.1005622.s009.pdf]

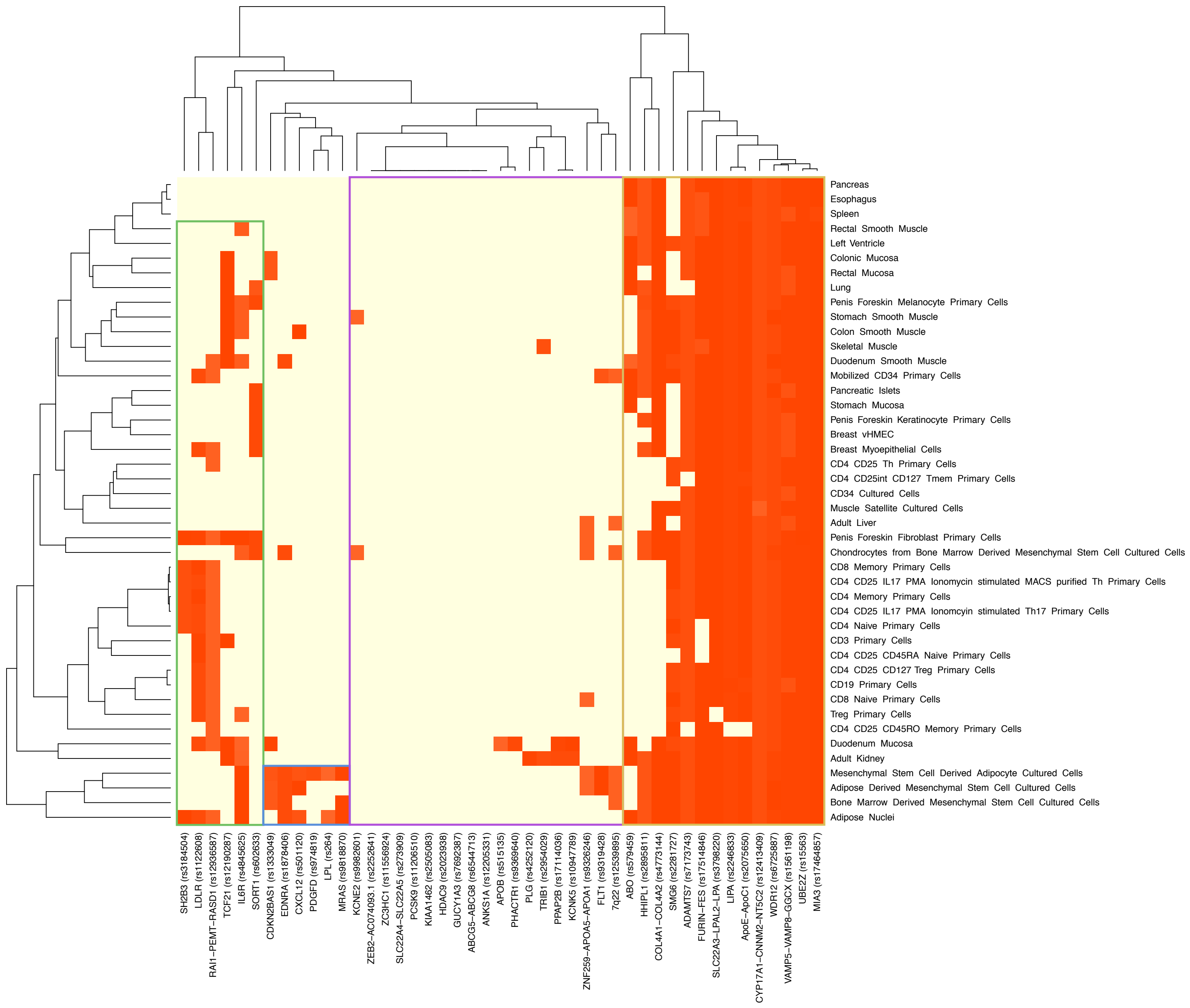

Supplement: S10 Fig — We mapped 45 MI/CAD GWAS SNPs, as well as SNPs in high linkage disequilibrium (r 2≥0.8), to H3K4me3 in different cell types. Hierarchical clustering was based on the presence or absence of a SNP residing in H3K4me3 in different cell types and was performed using the heatmap function in the R Project for Statistical Computing. We observed unique patterns between the different GWAS loci and cell types. MI, myocardial infarction; CAD, coronary artery disease; GWAS, genome-wide association study; SNP, single nucleotide polymorphism. (PDF) [file pgen.1005622.s010.pdf]

# Connectivity among 45 loci proteins (DAPPLE)

Adipose Nuclei (P=0.002, FDR P=0.049)

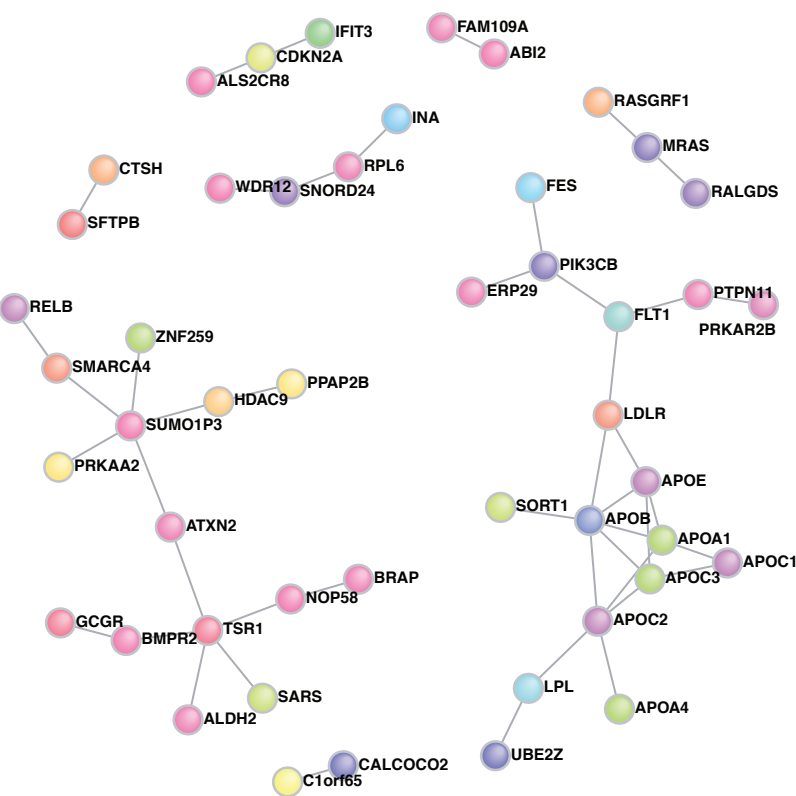

MSC Derived Adipocyte (P=0.001, FDR P=0.049)

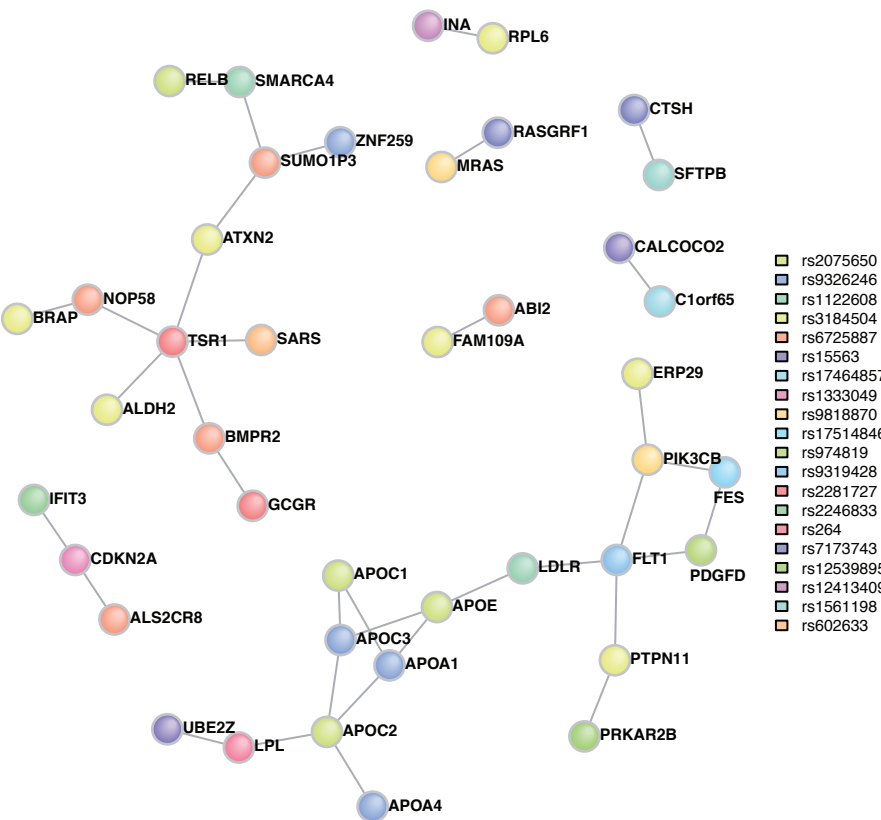

Supplement: S11 Fig — We tested for direct connectivity of genes in GWAS loci in specific cell types. We tested 45 MI/CAD GWAS SNPs, in addition to SNPs in high linkage disequilibrium (r 2≥0.8) (same SNP set in enrichment analysis), that overlapped with the three histone marks (H3K4me3, H3K9ac, H3K27ac) in a specific cell type. SNPs that only overlap the three histone marks in adipose nuclei and mesenchymal stem cell (MSC) derived adipocyte cell types were tested. DAPPLE [51] was utilized to test for direct connectivity in protein-protein interaction (PPI) networks. Gene regulatory regions were defined as within 110 kb upstream of transcription start site and 40 kb downstream of transcription end site of each of the 45 lead SNPs or tag SNPs were included in the analysis. We tested each variant set 1,000 times to obtain empirical significance for the observed connectivity compared with the expected connectivity. We observed high direct connectivity in a PPI network comprised of known lipid genes (for example, apolipoprotein E [APOE], apolipoprotein C3 [APOC3], low-density lipoprotein receptor [LDLR]) in adipose nuclei and MSC derived adipocyte cell types. (PDF) [file pgen.1005622.s011.pdf]
