# Supplementary material for: Disproportionate Contributions of Select Genomic Compartments and Cell Types to Genetic Risk for Coronary Artery Disease
Source: PLoS Genet. 2015 Oct 28;11(10):e1005622. doi: 10.1371/journal.pgen.1005622 (PMC4625039; doi:10.1371/journal.pgen.1005622)
Supplement: S6 Fig — We performed polygenic risk score and heritability analyses to test for cell type specific effects on the genetic risk for MI/CAD. Analyses were conducted on SNPs residing in eight active chromatin states inferred by ChromHMM [15] that were present in the different cell types. (A) Polygenic risk score analysis. We performed polygenic risk score association analysis on SNPs with MIGen discovery association P<0.05. Negative logarithm of P values from association testing of the polygenic risk score performed in the WTCCC CAD was shown. Cell types were sorted based on the strength of polygenic association. Orange vertical line represents a significant level with 5% alpha error. (B) Heritability analysis. Heritability analysis was performed within chromatin states in the MIGen study. Each point in the plot represents the variance in liability generated from a joint model involving two variance components using the Genome-wide Complex Trait Analysis software [22,23]. The two variance components include 1) SNPs in the specified chromatin state that was present in the indicated cell type and 2) all other SNPs outside of these regions. The variance in liability is an estimate from the ratio of genetic variance to phenotypic variance for the specified variance component (i.e. the specified variance component is all SNPs within the specified chromatin state) whereas the P value is from the likelihood ratio test of a reduce model with the specified genetic variance component dropped from the full model, from the restricted maximum likelihood method in the Genome-wide Complex Trait Analysis software [22,23]. MI, myocardial infarction; CAD, coronary artery disease; SNP, single nucleotide polymorphism. (PDF) [file pgen.1005622.s006.pdf]

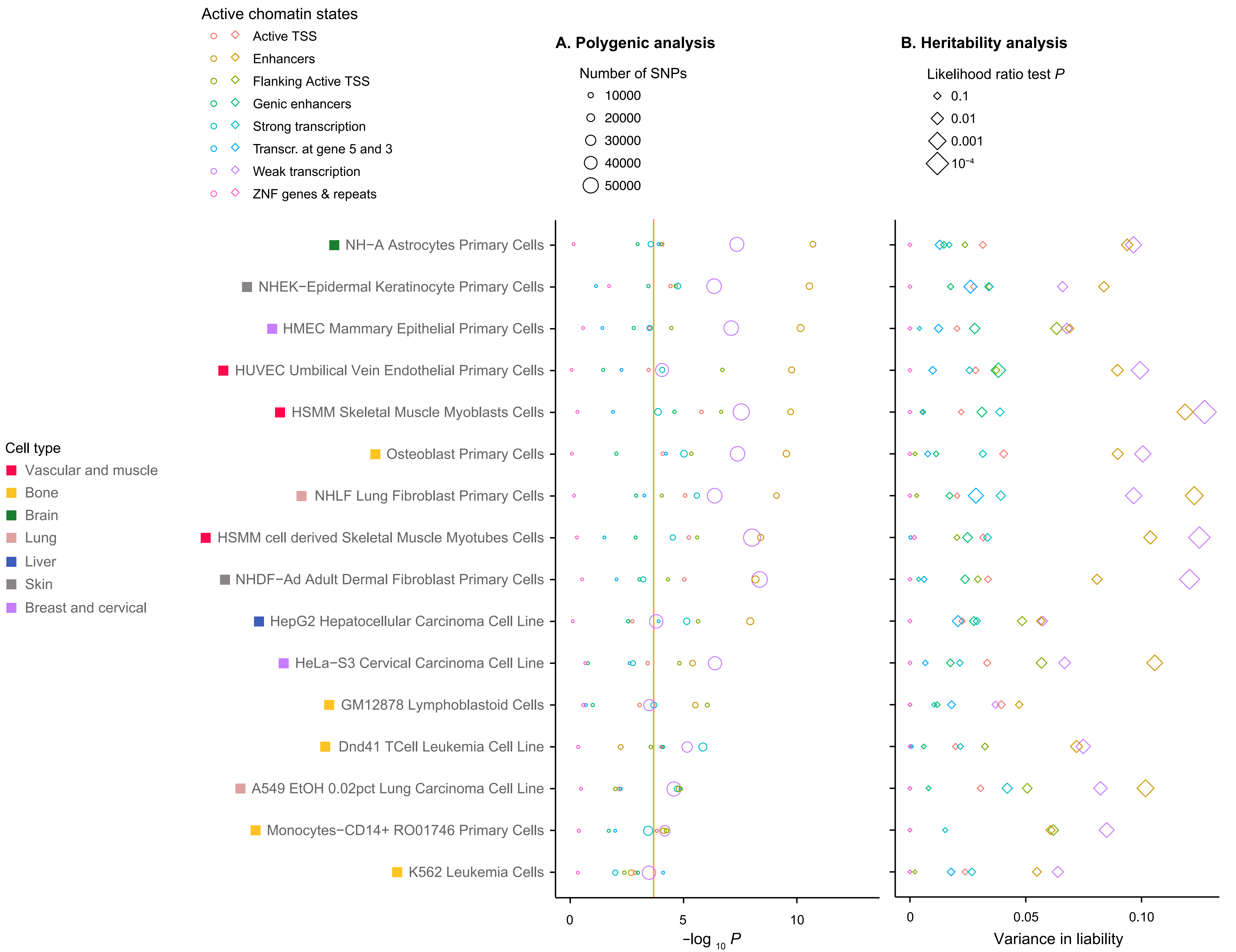

Active chomatin states
Active TSS
Enhancers
Flanking Active TSS
Genic enhancers
Strong transcription
Transcr. at gene 5 and 3
Weak transcription
ZNF genes & repeats
A. Polygenic analysis
Number of SNPs
10000
20000
30000
40000
50000
B. Heritability analysis
Likelihood ratio test P
0.1
0.01
0.001
10⁻⁴
Cell type
Vascular and muscle
Bone
Brain
Lung
Liver
Skin
Breast and cervical
NH−A Astrocytes Primary Cells
NHEK−Epidermal Keratinocyte Primary Cells
HMEC Mammary Epithelial Primary Cells
HUVEC Umbilical Vein Endothelial Primary Cells
HSMM Skeletal Muscle Myoblasts Cells
Osteoblast Primary Cells
NHLF Lung Fibroblast Primary Cells
HSMM cell derived Skeletal Muscle Myotubes Cells
NHDF−Ad Adult Dermal Fibroblast Primary Cells
HepG2 Hepatocellular Carcinoma Cell Line
HeLa−S3 Cervical Carcinoma Cell Line
GM12878 Lymphoblastoid Cells
Dnd41 TCell Leukemia Cell Line
A549 EtOH 0.02pct Lung Carcinoma Cell Line
Monocytes−CD14+ RO01746 Primary Cells
K562 Leukemia Cells
0
5
10
−log 10 P
0
0.05
0.10
Variance in liability
