# Supplementary material for: Disproportionate Contributions of Select Genomic Compartments and Cell Types to Genetic Risk for Coronary Artery Disease
Source: PLoS Genet. 2015 Oct 28;11(10):e1005622. doi: 10.1371/journal.pgen.1005622 (PMC4625039; doi:10.1371/journal.pgen.1005622)
Supplement: S1 Table — (DOCX) [file pgen.1005622.s012.docx]

**S1 table. Functional categorization of variants**

| **Functional compartments** | **Definition** |
| --- | --- |
| Genic coding variants | Variants that code amino acid sequence within ±10 (20, or 50) kilobases of the 3′ or 5′ untranslated regions of a gene |
| Genic noncoding variants | Variants that do not code amino acid sequence within ±10 (20, or 50) kilobases of the 3′ or 5′ untranslated regions of a gene |
| Intergenic variants | Variants that are beyond ±10 (20, or 50) kilobases of the 3′ or 5′ untranslated regions of a gene |
