# Supplementary material for: Disproportionate Contributions of Select Genomic Compartments and Cell Types to Genetic Risk for Coronary Artery Disease
Source: PLoS Genet. 2015 Oct 28;11(10):e1005622. doi: 10.1371/journal.pgen.1005622 (PMC4625039; doi:10.1371/journal.pgen.1005622)
Supplement: S2 Table — (DOCX) [file pgen.1005622.s013.docx]

**S2 table. Heritability of MI/CAD explained by three genomic compartment sets (10 kilobases window for genic regions).** We calculated the SNP-heritability in three genomic compartment sets for MI/CAD in a meta-analysis of the MIGen and WTCCC CAD studies using the Genome-wide Complex Trait Analysis (GCTA) software. We observed increased enrichment in variance in both “genic coding” and “genic noncoding” regions.
**A. Meta-analysis**

| **Genomic compartments** | **Variance^1^** | **V-SE^1^** | ***V-P*^1^** | **Number of SNPs** | **% Variance of total** | **% SNPs of total** | **Enrichment of variance^2^** | **Deviation from expected variance *P*^3^** |
| --- | --- | --- | --- | --- | --- | --- | --- | --- |
| Genic coding | 0.042 | 0.023 | 0.07 | 37,142 | 10.0 | 0.5 | 19.1 | 0.088 |
| Genic noncoding | 0.25 | 0.041 | 1×10^−9^ | 3,355,483 | 58.9 | 47.3 | 1.2 | 0.23 |
| Intergenic | 0.13 | 0.034 | 0.0001 | 3,703,319 | 31.1 | 52.2 | 0.6 | 0.0089 |
| Whole genome as sum | 0.42 |  |  | 7,095,944 | 100.0 | 100.0 | 1.0 |  |

**B. MIGen**

| **Genomic compartments** | **Variance^1^** | **V-SE^1^** | ***V-P*^1^** | **Number of SNPs** | **% Variance of total** | **% SNPs of total** | **Enrichment of variance^2^** | **Deviation from expected variance *P*^3^** |
| --- | --- | --- | --- | --- | --- | --- | --- | --- |
| Genic coding | 0.042 | 0.030 | 0.08 | 37,210 | 10.0 | 0.5 | 19.0 | 0.18 |
| Genic noncoding | 0.25 | 0.053 | 6×10^−7^ | 3,355,483 | 59.4 | 47.3 | 1.3 | 0.33 |
| Intergenic | 0.13 | 0.045 | 0.0015 | 3,702,939 | 30.6 | 52.2 | 0.6 | 0.04 |
| Whole genome as sum | 0.42 |  |  | 7,095,632 | 100.0 | 100.0 | 1.0 |  |

**C. WTCCC CAD**

| **Genomic compartments** | **Variance^1^** | **V-SE^1^** | ***V-P*^1^** | **Number of SNPs** | **% Variance of total** | **% SNPs of total** | **Enrichment of variance^2^** | **Deviation from expected variance *P*^3^** |
| --- | --- | --- | --- | --- | --- | --- | --- | --- |
| Genic coding | 0.042 | 0.037 | 0.14 | 37,035 | 10.1 | 0.5 | 19.4 | 0.29 |
| Genic noncoding | 0.24 | 0.064 | 8×10^−5^ | 3,355,483 | 58.2 | 47.3 | 1.2 | 0.48 |
| Intergenic | 0.13 | 0.052 | 0.0047 | 3,703,834 | 31.7 | 52.2 | 0.6 | 0.11 |
| Whole genome as sum | 0.41 |  |  | 7,096,352 | 100.0 | 100.0 | 1.0 |  |

Heritability estimates were inferred independently first in MIGen and WTCCC CAD from a single model involving three variance components (“genic coding”, “genic noncoding” and “intergenic”) using the GCTA software [[21](#_ENREF_21),[22](#_ENREF_22)]. Heritability estimates shown here are from a meta-analysis of the Variance and standard error (V-SE) from these models using as weights the inverse variance from these models. ^1^Variance and V-SE are estimates from the ratio of genetic variance to phenotypic variance for the specified variance component whereas the *P* value (V-P) is from the likelihood ratio test of a reduce model with the specified genetic variance component dropped from the full model, from the restricted maximum likelihood method in the GCTA software [[21](#_ENREF_21),[22](#_ENREF_22)]. ^2^Enrichment of variance was calculated as the % variance of total divided by % SNPs of total. MI, myocardial infarction; CAD, coronary artery disease; SNP, single nucleotide polymorphism. ^3^*P* value from difference in the observed variance minus the expected variance (variance of whole genome as sum multiplied by % SNPs of total). Genic coding, variants that code amino acid sequence within ±10 kilobases of the 3′ or 5′ untranslated regions of a gene. Genic noncoding, variants that do not code amino acid sequence within ±10 kilobases of the 3′ or 5′ untranslated regions of a gene. Intergenic, variants that are beyond ±10 kilobases of the 3′ or 5′ untranslated regions of a gene.
