# Supplementary material for: Disproportionate Contributions of Select Genomic Compartments and Cell Types to Genetic Risk for Coronary Artery Disease
Source: PLoS Genet. 2015 Oct 28;11(10):e1005622. doi: 10.1371/journal.pgen.1005622 (PMC4625039; doi:10.1371/journal.pgen.1005622)
Supplement: S3 Table — (DOCX) [file pgen.1005622.s014.docx]

**S3 table. Heritability of MI/CAD explained by three genomic compartment sets (20 kilobases window for genic regions).** We calculated the SNP-heritability in three genomic compartment sets for MI/CAD in a meta-analysis of the MIGen and WTCCC CAD studies using the Genome-wide Complex Trait Analysis (GCTA) software. We observed increased enrichment in variance in both “genic coding” and “genic noncoding” regions.
**A. Meta-analysis**

| **Genomic compartments** | **Variance^1^** | **V-SE^1^** | ***V-P*^1^** | **Number of SNPs** | **% Variance of total** | **% SNPs of total** | **Enrichment of variance^2^** | **Deviation from expected variance *P*^3^** |
| --- | --- | --- | --- | --- | --- | --- | --- | --- |
| Genic coding | 0.044 | 0.023 | 0.06 | 37,144 | 10.4 | 0.5 | 19.8 | 0.074 |
| Genic noncoding | 0.27 | 0.042 | 7×10^−11^ | 3,739,851 | 64.9 | 52.7 | 1.2 | 0.22 |
| Intergenic | 0.10 | 0.031 | 0.0009 | 3,319,465 | 24.7 | 46.8 | 0.5 | 0.0032 |
| Whole genome as sum | 0.42 |  |  | 7,096,460 | 100.0 | 100.0 | 1.0 |  |

**B. MIGen**

| **Genomic compartments** | **Variance^1^** | **V-SE^1^** | ***V-P*^1^** | **Number of SNPs** | **% Variance of total** | **% SNPs of total** | **Enrichment of variance^2^** | **Deviation from expected variance *P*^3^** |
| --- | --- | --- | --- | --- | --- | --- | --- | --- |
| Genic coding | 0.048 | 0.029 | 0.046 | 37,210 | 11.3 | 0.5 | 22 | 0.12 |
| Genic noncoding | 0.27 | 0.054 | 2×10^−7^ | 3,739,851 | 64.0 | 52.7 | 1.2 | 0.37 |
| Intergenic | 0.10 | 0.041 | 0.0048 | 3,319,465 | 24.6 | 46.8 | 0.5 | 0.02 |
| Whole genome as sum | 0.42 |  |  | 7,096,526 | 100.0 | 100.0 | 1.0 |  |

**C. WTCCC CAD**

| **Genomic compartments** | **Variance^1^** | **V-SE^1^** | ***V-P*^1^** | **Number of SNPs** | **% Variance of total** | **% SNPs of total** | **Enrichment of variance^2^** | **Deviation from expected variance *P*^3^** |
| --- | --- | --- | --- | --- | --- | --- | --- | --- |
| Genic coding | 0.036 | 0.038 | 0.18 | 37,035 | 8.8 | 0.5 | 16.8 | 0.37 |
| Genic noncoding | 0.27 | 0.066 | 2×10^−5^ | 3,739,851 | 66.3 | 52.7 | 1.3 | 0.40 |
| Intergenic | 0.10 | 0.048 | 0.0135 | 3,319,465 | 25.0 | 46.8 | 0.5 | 0.06 |
| Whole genome as sum | 0.41 |  |  | 7,096,351 | 100.0 | 100.0 | 1.0 |  |

Heritability estimates were inferred independently first in MIGen and WTCCC CAD from a single model involving three variance components (“genic coding”, “genic noncoding” and “intergenic”) using the GCTA software [[21](#_ENREF_21),[22](#_ENREF_22)]. Heritability estimates shown here are from a meta-analysis of the Variance and standard error (V-SE) from these models using as weights the inverse variance from these models. ^1^Variance and V-SE are estimates from the ratio of genetic variance to phenotypic variance for the specified variance component whereas the *P* value (V-P) is from the likelihood ratio test of a reduce model with the specified genetic variance component dropped from the full model, from the restricted maximum likelihood method in the GCTA software [[21](#_ENREF_21),[22](#_ENREF_22)]. ^2^Enrichment of variance was calculated as the % variance of total divided by % SNPs of total. MI, myocardial infarction; CAD, coronary artery disease; SNP, single nucleotide polymorphism. ^3^*P* value from difference in the observed variance minus the expected variance (variance of whole genome as sum multiplied by % SNPs of total). Genic coding, variants that code amino acid sequence within ±20 kilobases of the 3′ or 5′ untranslated regions of a gene. Genic noncoding, variants that do not code amino acid sequence within ±20 kilobases of the 3′ or 5′ untranslated regions of a gene. Intergenic, variants that are beyond ±20 kilobases of the 3′ or 5′ untranslated regions of a gene.
