# Supplementary material for: Disproportionate Contributions of Select Genomic Compartments and Cell Types to Genetic Risk for Coronary Artery Disease
Source: PLoS Genet. 2015 Oct 28;11(10):e1005622. doi: 10.1371/journal.pgen.1005622 (PMC4625039; doi:10.1371/journal.pgen.1005622)
Supplement: S4 Table — (DOCX) [file pgen.1005622.s015.docx]

**S4 table. Heritability of MI/CAD explained by three genomic compartment sets (50 kilobases window for genic regions).** We calculated the SNP-heritability in three genomic compartment sets for MI/CAD in a meta-analysis of the MIGen and WTCCC CAD studies using the Genome-wide Complex Trait Analysis (GCTA) software. We observed increased enrichment in variance in both “genic coding” and “genic noncoding” regions.
**A. Meta-analysis**

| **Genomic compartments** | **Variance^1^** | **V-SE^1^** | ***V-P*^1^** | **Number of SNPs** | **% Variance of total** | **% SNPs of total** | **Enrichment of variance^2^** | **Deviation from expected variance *P*^3^** |
| --- | --- | --- | --- | --- | --- | --- | --- | --- |
| Genic coding | 0.045 | 0.023 | 0.05 | 37,144 | 10.6 | 0.5 | 20.3 | 0.06 |
| Genic noncoding | 0.30 | 0.043 | 3E-12 | 4,421,285 | 71.5 | 62.3 | 1.1 | 0.37 |
| Intergenic | 0.08 | 0.027 | 0.0055 | 2,638,027 | 17.9 | 37.2 | 0.5 | 0.0026 |
| Whole genome as sum | 0.42 |  |  | 7,096,456 | 100.0 | 100.0 | 1.0 |  |

**B. MIGen**

| **Genomic compartments** | **Variance^1^** | **V-SE^1^** | ***V-P*^1^** | **Number of SNPs** | **% Variance of total** | **% SNPs of total** | **Enrichment of variance^2^** | **Deviation from expected variance *P*^3^** |
| --- | --- | --- | --- | --- | --- | --- | --- | --- |
| Genic coding | 0.052 | 0.029 | 0.03 | 37,210 | 12.3 | 0.5 | 24 | 0.08 |
| Genic noncoding | 0.29 | 0.056 | 1E-07 | 4,421,285 | 67.6 | 62.3 | 1.1 | 0.68 |
| Intergenic | 0.09 | 0.036 | 0.0075 | 2,638,027 | 20.0 | 37.2 | 0.5 | 0.04 |
| Whole genome as sum | 0.42 |  |  | 7,096,522 | 100.0 | 100.0 | 1.0 |  |

**C. WTCCC CAD**

| **Genomic compartments** | **Variance^1^** | **V-SE^1^** | ***V-P*^1^** | **Number of SNPs** | **% Variance of total** | **% SNPs of total** | **Enrichment of variance^2^** | **Deviation from expected variance *P*^3^** |
| --- | --- | --- | --- | --- | --- | --- | --- | --- |
| Genic coding | 0.033 | 0.037 | 0.20 | 37,035 | 7.8 | 0.5 | 15.0 | 0.41 |
| Genic noncoding | 0.32 | 0.068 | 1E-06 | 4,421,285 | 77.3 | 62.3 | 1.2 | 0.36 |
| Intergenic | 0.06 | 0.042 | 0.0620 | 2,638,027 | 14.9 | 37.2 | 0.4 | 0.03 |
| Whole genome as sum | 0.42 |  |  | 7,096,347 | 100.0 | 100.0 | 1.0 |  |

Heritability estimates were inferred independently first in MIGen and WTCCC CAD from a single model involving three variance components (“genic coding”, “genic noncoding” and “intergenic”) using the GCTA software [[21](#_ENREF_21),[22](#_ENREF_22)]. Heritability estimates shown here are from a meta-analysis of the Variance and standard error (V-SE) from these models using as weights the inverse variance from these models. ^1^Variance and V-SE are estimates from the ratio of genetic variance to phenotypic variance for the specified variance component whereas the *P* value (V-P) is from the likelihood ratio test of a reduce model with the specified genetic variance component dropped from the full model, from the restricted maximum likelihood method in the GCTA software [[21](#_ENREF_21),[22](#_ENREF_22)]. ^2^Enrichment of variance was calculated as the % variance of total divided by % SNPs of total. MI, myocardial infarction; CAD, coronary artery disease; SNP, single nucleotide polymorphism. ^3^*P* value from difference in the observed variance minus the expected variance (variance of whole genome as sum multiplied by % SNPs of total). Genic coding, variants that code amino acid sequence within ±50 kilobases of the 3′ or 5′ untranslated regions of a gene. Genic noncoding, variants that do not code amino acid sequence within ±50 kilobases of the 3′ or 5′ untranslated regions of a gene. Intergenic, variants that are beyond ±50 kilobases of the 3′ or 5′ untranslated regions of a gene.
