# Supplementary material for: Disproportionate Contributions of Select Genomic Compartments and Cell Types to Genetic Risk for Coronary Artery Disease
Source: PLoS Genet. 2015 Oct 28;11(10):e1005622. doi: 10.1371/journal.pgen.1005622 (PMC4625039; doi:10.1371/journal.pgen.1005622)
Supplement: S5 Table — (DOCX) [file pgen.1005622.s016.docx]

**S5 table. Heritability with a prevalence of 3% of MI/CAD explained by three genomic compartment sets.** We calculated the SNP-heritability in three genomic compartment sets for MI/CAD in the MIGen study using the Genome-wide Complex Trait Analysis (GCTA) software. We observed increased enrichment in variance in both “genic coding” and “genic noncoding” regions.
**A. 10 kilobases window for genic regions**

| **Genomic compartments** | **Variance^1^** | **V-SE^1^** | ***V-P*^1^** | **Number of SNPs** | **% Variance of total** | **% SNPs of total** | **Enrichment of variance^2^** | **Deviation from expected variance *P*^3^** |
| --- | --- | --- | --- | --- | --- | --- | --- | --- |
| Genic coding | 0.034 | 0.024 | 0.08 | 37,210 | 10.0 | 0.5 | 19.0 | 0.18 |
| Genic noncoding | 0.206 | 0.043 | 6×10^−7^ | 3,355,483 | 59.4 | 47.3 | 1.3 | 0.33 |
| Intergenic | 0.106 | 0.036 | 0.0015 | 3,702,939 | 30.6 | 52.2 | 0.6 | 0.04 |
| Whole genome as sum | 0.347 |  |  | 7,095,632 | 100.0 | 100.0 | 1.0 |  |

**B. 20 kilobases window for genic regions**

| **Genomic compartments** | **Variance^1^** | **V-SE^1^** | ***V-P*^1^** | **Number of SNPs** | **% Variance of total** | **% SNPs of total** | **Enrichment of variance^2^** | **Deviation from expected variance *P*^3^** |
| --- | --- | --- | --- | --- | --- | --- | --- | --- |
| Genic coding | 0.039 | 0.024 | 0.046 | 37,210 | 11.3 | 0.5 | 22 | 0.12 |
| Genic noncoding | 0.221 | 0.044 | 2×10^−7^ | 3,739,851 | 64.0 | 52.7 | 1.2 | 0.37 |
| Intergenic | 0.085 | 0.034 | 0.0048 | 3,319,465 | 24.6 | 46.8 | 0.5 | 0.02 |
| Whole genome as sum | 0.346 |  |  | 7,096,526 | 100.0 | 100.0 | 1.0 |  |

**C. 50 kilobases window for genic regions**

| **Genomic compartments** | **Variance^1^** | **V-SE^1^** | ***V-P*^1^** | **Number of SNPs** | **% Variance of total** | **% SNPs of total** | **Enrichment of variance^2^** | **Deviation from expected variance *P*^3^** |
| --- | --- | --- | --- | --- | --- | --- | --- | --- |
| Genic coding | 0.043 | 0.024 | 0.03 | 37,210 | 12.3 | 0.5 | 24 | 0.08 |
| Genic noncoding | 0.234 | 0.046 | 1E-07 | 4,421,285 | 67.6 | 62.3 | 1.1 | 0.68 |
| Intergenic | 0.069 | 0.029 | 0.0075 | 2,638,027 | 20.0 | 37.2 | 0.5 | 0.04 |
| Whole genome as sum | 0.347 |  |  | 7,096,522 | 100.0 | 100.0 | 1.0 |  |

Heritability estimates were inferred from a single model involving three variance components (“genic coding”, “genic noncoding” and “intergenic”) using the GCTA software [[21](#_ENREF_21),[22](#_ENREF_22)]. ^1^Variance and V-SE are estimates from the ratio of genetic variance to phenotypic variance for the specified variance component whereas the *P* value (V-P) is from the likelihood ratio test of a reduce model with the specified genetic variance component dropped from the full model, from the restricted maximum likelihood method in the GCTA software [[21](#_ENREF_21),[22](#_ENREF_22)]. ^2^Enrichment of variance was calculated as the % variance of total divided by % SNPs of total. MI, myocardial infarction; CAD, coronary artery disease; SNP, single nucleotide polymorphism. ^3^*P* value from difference in the observed variance minus the expected variance (variance of whole genome as sum multiplied by % SNPs of total). Genic coding, variants that code amino acid sequence within ±10 (20, or 50) kilobases of the 3′ or 5′ untranslated regions of a gene. Genic noncoding, variants that do not code amino acid sequence within ±10 (20, or 50) kilobases of the 3′ or 5′ untranslated regions of a gene. Intergenic, variants that are beyond ±10 (20, or 50) kilobases of the 3′ or 5′ untranslated regions of a gene.
