# Supplementary material for: Disproportionate Contributions of Select Genomic Compartments and Cell Types to Genetic Risk for Coronary Artery Disease
Source: PLoS Genet. 2015 Oct 28;11(10):e1005622. doi: 10.1371/journal.pgen.1005622 (PMC4625039; doi:10.1371/journal.pgen.1005622)
Supplement: S6 Table — (DOCX) [file pgen.1005622.s017.docx]

**S6 table. Cell-type specific protein-protein interaction network among neighboring genes of 45 MI/CAD GWAS loci on three histone marks**

| **Cell** | ***P* for direct edges count** | ***P* for seed direct degrees mean** | ***P* for seed indirect degrees mean** | ***P* for CI degrees mean** |
| --- | --- | --- | --- | --- |
| Mesenchymal Stem Cell Derived Adipocyte Cultured Cells | 0.001 | 0.042 | 0.666 | 0.158 |
| Adipose Nuclei | 0.002 | 0.046 | 0.555 | 0.336 |
| Adult Liver | 0.009 | 0.008 | 0.861 | 0.056 |
| CD8 Naive Primary Cells | 0.011 | 0.035 | 0.439 | 0.051 |
| Chondrocytes from Bone Marrow Derived Mesenchymal Stem Cell Cultured Cells | 0.016 | 0.251 | 0.618 | 0.161 |
| CD4 CD25 IL17 PMA Ionomcyin stimulated Th17 Primary Cells | 0.022 | 0.039 | 0.624 | 0.060 |
| CD4 CD25 IL17 PMA Ionomycin stimulated MACS purified Th Primary Cells | 0.029 | 0.050 | 0.641 | 0.061 |
| Spleen | 0.029 | 0.257 | 0.291 | 0.207 |
| Bone Marrow Derived Mesenchymal Stem Cell Cultured Cells | 0.037 | 0.155 | 0.857 | 0.127 |
| CD4 Naive Primary Cells | 0.038 | 0.209 | 0.298 | 0.309 |
| Skeletal Muscle | 0.049 | 0.144 | 0.718 | 0.426 |
| Penis Foreskin Fibroblast Primary Cells | 0.050 | 0.076 | 0.878 | 0.179 |
| Adipose Derived Mesenchymal Stem Cell Cultured Cells | 0.053 | 0.075 | 0.958 | 0.074 |
| CD4 CD25 CD45RA Naive Primary Cells | 0.056 | 0.211 | 0.476 | 0.405 |
| CD8 Memory Primary Cells | 0.072 | 0.346 | 0.066 | 0.188 |
| CD4 CD25 CD45RO Memory Primary Cells | 0.079 | 0.155 | 0.427 | 0.432 |
| Right Atrium | 0.085 | 0.260 | 0.873 | 0.233 |
| CD4 Memory Primary Cells | 0.097 | 0.239 | 0.418 | 0.392 |
| Duodenum Mucosa | 0.102 | 0.395 | 0.311 | 0.238 |
| Muscle Satellite Cultured Cells | 0.102 | 0.428 | 0.804 | 0.156 |
| Penis Foreskin Keratinocyte Primary Cells | 0.115 | 0.637 | 0.146 | 0.172 |
| CD3 Primary Cells | 0.126 | 0.347 | 0.151 | 0.312 |
| CD4 CD25 Th Primary Cells | 0.153 | 0.260 | 0.617 | 0.463 |
| Rectal Mucosa | 0.164 | 0.324 | 0.502 | 0.210 |
| Lung | 0.187 | 0.423 | 0.580 | 0.140 |
| Colon Smooth Muscle | 0.199 | 0.313 | 0.645 | 0.383 |
| Duodenum Smooth Muscle | 0.206 | 0.343 | 0.572 | 0.118 |
| Colonic Mucosa | 0.208 | 0.369 | 0.590 | 0.189 |
| Sigmoid Colon | 0.215 | 0.484 | 0.370 | 0.209 |
| CD19 Primary Cells | 0.234 | 0.359 | 0.258 | 0.437 |
| CD4 CD25 CD127 Treg Primary Cells | 0.242 | 0.382 | 0.253 | 0.423 |
| Pancreatic Islets | 0.262 | 0.447 | 0.288 | 0.312 |
| Treg Primary Cells | 0.268 | 0.376 | 0.425 | 0.502 |
| Esophagus | 0.278 | 0.503 | 0.469 | 0.307 |
| Penis Foreskin Melanocyte Primary Cells | 0.313 | 0.304 | 0.804 | 0.533 |
| Stomach Smooth Muscle | 0.354 | 0.222 | 0.902 | 0.290 |
| Mobilized CD34 Primary Cells | 0.373 | 0.722 | 0.210 | 0.452 |
| Breast Fibroblast Primary Cells | 0.396 | 0.568 | 0.766 | 0.312 |
| Adult Kidney | 0.408 | 0.359 | 0.604 | 0.141 |
| Peripheral Blood Mononuclear Primary Cells | 0.437 | 0.424 | 0.584 | 0.469 |
| CD4 CD25int CD127 Tmem Primary Cells | 0.590 | 0.196 | 0.636 | 0.329 |
| Left Ventricle | 0.617 | 0.654 | 0.323 | 0.108 |
| Stomach Mucosa | 0.658 | 0.561 | 0.675 | 0.207 |
| Rectal Smooth Muscle | 0.677 | 0.667 | 0.559 | 0.154 |
| Fetal Lung | 0.728 | 0.285 | 0.898 | 0.426 |
| Breast Myoepithelial Cells | 0.794 | 0.521 | 0.867 | 0.365 |
| Pancreas | 0.846 | 0.997 | 0.457 | 0.099 |
| CD34 Cultured Cells | 0.860 | 0.966 | 0.860 | 0.285 |
| Breast vHMEC | 0.941 | 0.996 | 0.875 | 0.361 |

MI, myocardial infarction; CAD, coronary artery disease; GWAS, genome-wide association study; CI, common interactor.
